# Molecular characteristics, clonal relatedness and surgical outcomes of pulmonary mixed invasive mucinous and non-mucinous adenocarcinoma: a retrospective cohort study

**DOI:** 10.1186/s43556-026-00460-1

**Published:** 2026-05-09

**Authors:** Xinyi Shi, Yang Wang, Nan Yao, Bowen Xue, Lei Guo, Liming Xu, Changbin Zhu, Guiping Qin, Jianming Ying, Yutao Liu, Weihua Li

**Affiliations:** 1 Department of Pathology, State Key Laboratory of Molecular Oncology, National Cancer Center/National Clinical Research Center for Cancer/Cancer Hospital, Chinese Academy of Medical Sciences and Peking Union Medical College, Beijing, China; 2 Department of Pathology, Liangxiang Hospital of Beijing Fangshan District, Beijing, China; 3 Department of Medical Oncology, National Cancer Center/National Clinical Research Center for Cancer/Cancer Hospital, Chinese Academy of Medical Sciences and Peking Union Medical College, Beijing, China; 4 Amoy Diagnostics Co., Ltd., Xiamen, China

**Keywords:** Mixed invasive mucinous and non-mucinous adenocarcinomas, Driver alterations, Tumor microenvironment, Clonal relatedness, Tumor heterogeneity, Surgical outcomes

## Abstract

Mixed invasive mucinous adenocarcinoma and non-mucinous adenocarcinoma (mixed IMA/NMA) is a rare subtype of lung adenocarcinoma (LUAD) with limited available data. This study aimed to comprehensively analyze the characteristics of this rare entity. A total of 738 surgical cases were enrolled, including 349 pure invasive mucinous adenocarcinoma (IMA), 61 mixed IMA/NMA and 328 pure non-mucinous adenocarcinoma (NMA) cases. Using amplification refractory mutation system, immunohistochemistry, and DNA-/RNA-based next-generation sequencing (DNA and RNA NGS), distinct molecular features were identified in mixed IMA/NMA cases compared with IMA and NMA cases, particularly in *EGFR*, *KRAS*, and *ALK* alterations and PD-L1 expression status. Paired analysis of the IMA and NMA components within mixed IMA/NMA cases using DNA and RNA NGS revealed one to three shared genomic alterations between the two components in the same tumor. However, significant differences were observed in the levels of cancer-associated fibroblasts, protumor cytokines, MHC-II, coactivation molecules, T cells, and effector cells between the components. Similarly, multiplex immunofluorescence assay demonstrated that immune cell infiltration, including CD4^+^ and CD8^+^ T cells, was significantly higher in the NMA components compared to the IMA components. Postoperative follow-up revealed no significant difference in disease-free survival (DFS) or overall survival (OS) between NMA and mixed IMA/NMA cases; however, both groups showed significantly shorter DFS (*P* = 0.011) and OS (*P* = 0.027) compared to IMA cases. Together, this study provides a comprehensive characterization of the molecular profiles, clonal relatedness, tumor heterogeneity, and surgical outcomes of mixed IMA/NMA, which may inform diagnostic and therapeutic strategies for this rare LUAD subtype.

**Supplementary Information:**

The online version contains supplementary material available at 10.1186/s43556-026-00460-1.

## Introduction

Lung cancer remains one of the most prevalent malignant tumors worldwide, and lung adenocarcinoma (LUAD) is the most common histological subtype [1, 2]. Based on histopathological features, LUAD can be classified into invasive mucinous adenocarcinoma (IMA) [3, 4], non-mucinous adenocarcinoma (NMA), and mixed invasive mucinous and non-mucinous adenocarcinoma (mixed IMA/NMA). NMA is the predominant form of LUAD, whereas IMA accounts for approximately 5% of LUAD cases [5, 6]. Mixed IMA/NMA, defined by the presence of more than 10% of both mucinous and non-mucinous components, represents a rare subtype of LUAD [7, 8].

The histological morphology of pure NMA primarily includes classic subtypes such as lepidic, acinar, papillary, micropapillary, and solid adenocarcinoma. In contrast, pure IMA exhibits distinct histomorphological features [9]. Clinically, compared to NMA, IMA more frequently occurs in the lower lobes of the lungs and is associated with multifocal lesions involving bilateral lungs [5, 10]. Current understanding of IMA’s molecular characteristics has largely relied on studies with limited sample sizes. A study representing the largest cohort of IMA patients to date (*n* = 200) analyzed tissue samples using sequential multi-platform molecular testing, including DNA-based next-generation sequencing (DNA NGS) and RNA NGS. This research revealed unique molecular profiles in IMA compared to NMA, characterized by frequent *KRAS* mutations, *NRG1* fusions, and *HER2* mutations [11]. However, research on the molecular characteristics of mixed IMA/NMA remains notably scarce.

Moreover, mixed IMA/NMA consists of two distinct components: the IMA component and the NMA component. Their clonal relationship and genomic heterogeneity remain unknown. Studies have explored the tumor immune microenvironment (TIME) characteristics of LUAD and revealed their close association with histological subtypes and specific genetic alterations [12]. However, these analyses have predominantly focused on NMA, leaving the immune microenvironment of IMA largely unexplored. Furthermore, it remains unclear whether the IMA and NMA components within the same mixed IMA/NMA case share similar TIME features or exhibit distinct characteristics. Additionally, emerging clinical data have focused on investigating the survival prognosis of NMA and IMA [13]. Nevertheless, there is still a lack of studies exploring the clinical outcomes of mixed IMA/NMA.

**Fig. 1:**
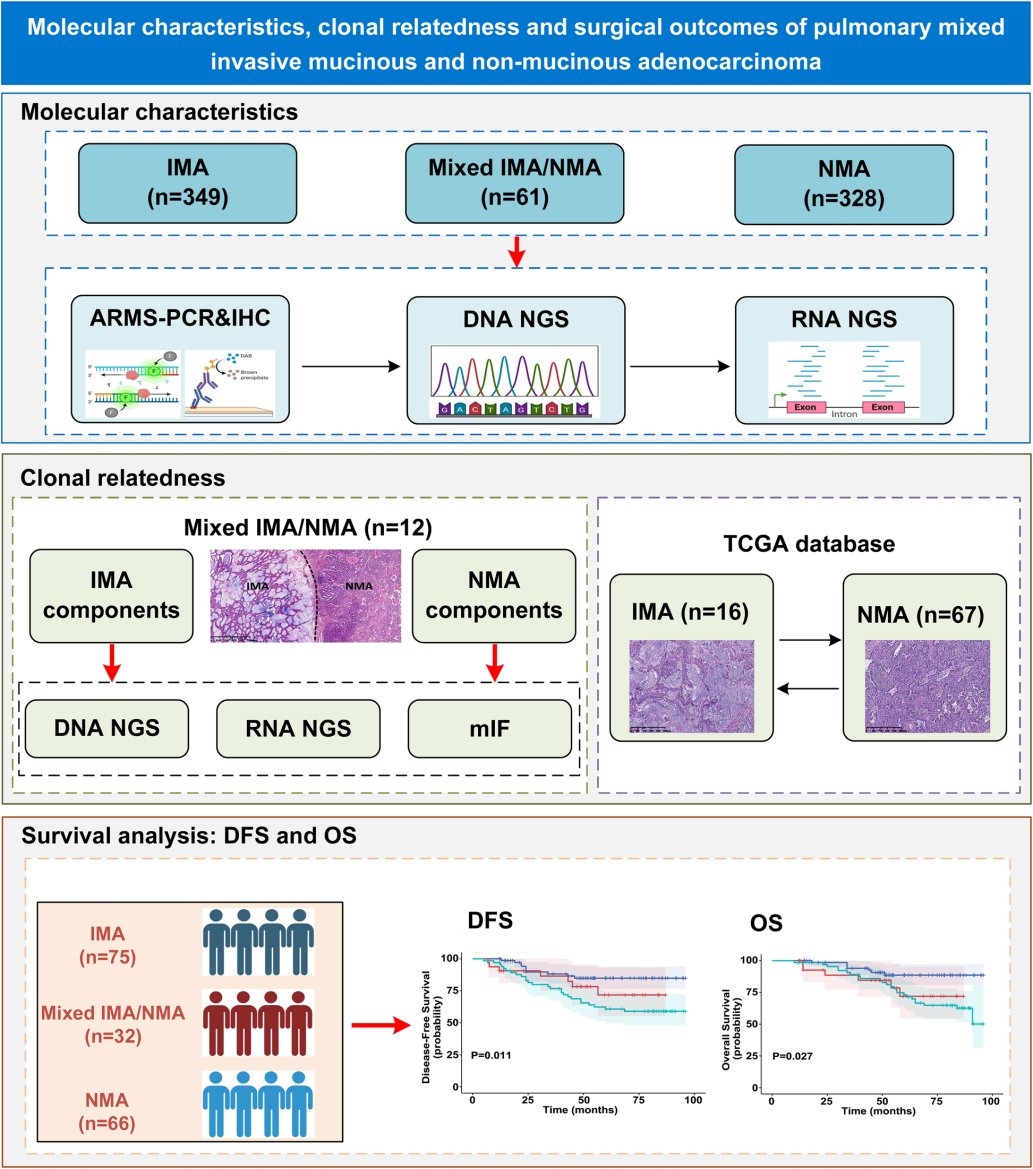
Schematic overview of the analysis of molecular characteristics, clonal relatedness and surgical outcomes in mixed IMA/NMA

In this study, we aim to investigate the clinicopathological features, molecular profiles, and survival outcomes of mixed IMA/NMA in comparison with pure IMA and NMA. Moreover, we sought to explore the clonal relatedness and intratumoral heterogeneity between the IMA and NMA components within mixed IMA/NMA (Fig. 1). In doing so, we hope to comprehensively analyze the characteristics of this rare LUAD subtype.

## Results

### Clinicopathological features of mixed IMA/NMA

Among the 738 LUAD cases, 349 were classified as IMA, 328 as NMA, and 61 as mixed IMA/NMA according to tumor pathological subtype. Clinicopathological characteristics were compared among the three groups (Table 1). No significant differences were observed in age distribution. However, mixed IMA/NMA was more prevalent in males (57.4% vs. 39.3% in IMA and 37.2% in NMA; *P* = 0.006), and was associated with a higher proportion of smoking history (37.7% vs. 24.4% in IMA and 30.5% in NMA; *P* = 0.047) and advanced clinical stage (39.3% vs. 11.2% in IMA and 10.4% in NMA; *P* < 0.001) compared to the other two groups.

**Table 1:** Clinical data of patients with LUAD of different subtypes

Patient	Total	IMA	Mixed IMA/NMA	NMA	*P* Value
Age					
≤ 60	402 (54.5%)	204 (58.5%)	33 (54.1%)	165 (50.3%)	0.104
> 60	336 (45.5%)	145 (41.6%)	28 (45.9%)	163 (49.7%)	
Sex					
Male	294 (39.8%)	137 (39.3%)	35 (57.4%)	122 (37.2%)	0.006
Female	444 (60.2%)	212 (60.7%)	26 (42.6%)	206 (62.8%)	
Stage					
I/II	641 (86.9%)	310(88.8%)	37(60.7%)	294(89.6%)	< 0.001
III/IV	97 (13.1%)	39(11.2%)	24(39.3%)	34(10.4%)	
Smoking					
Ex- or current	208 (28.2%)	85 (24.4%)	23 (37.7%)	100 (30.5%)	0.047
Never	530 (71.8%)	264 (75.6%)	38 (62.3%)	228 (69.5%)	
*EGFR* mutations					
Yes	259 (35.1%)	8 (2.3%)	14 (23.0%)	237 (72.3%)	< 0.001
No	479 (64.9%)	341 (97.7%)	47 (77.0%)	91 (27.7%)	
*KRAS* mutations					
Yes	200 (27.1%)	162 (46.4%)	15 (24.6%)	23 (7.0%)	< 0.001
No	538 (72.9%)	187 (53.6%)	46 (75.4%)	305 (93.0%)	
*ALK* fusions					
Yes	73 (9.9%)	48 (13.8%)	14 (23.0%)	11 (3.4%)	< 0.001
No	665 (90.1%)	301 (86.2%)	47 (77.0%)	317 (96.6%)	

### Molecular characteristics of mixed IMA/NMA

**Fig. 2:**
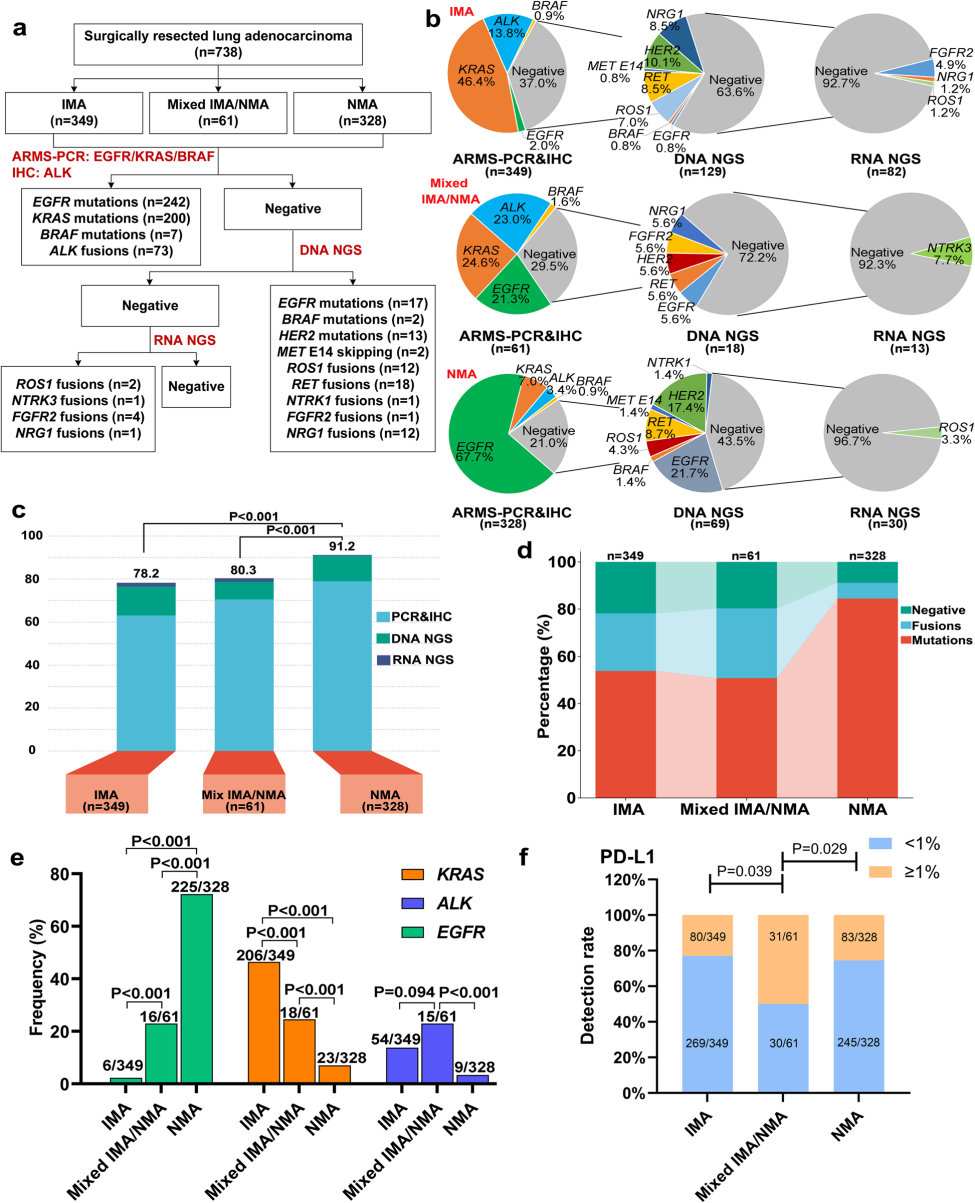
Molecular feature differences among IMA, mixed IMA/NMA, and NMA. **a** Flowchart of molecular testing across the IMA, mixed IMA/NMA, and NMA groups. **b** Driver alterations identified by sequential ARMS plus IHC, DNA NGS, and RNA NGS in the IMA, mixed IMA/NMA and NMA groups. **c** Proportion of driver alterations detected by ARMS plus IHC, DNA NGS, and RNA NGS across the three groups. **d** Proportion of patients with detected mutations and fusions across the three groups. **e** Incidence of *EGFR*, *KRAS*, and *ALK* alterations across the three groups. **f** Comparison of PD-L1 expression levels among the three patient groups

All 738 LUAD cases were initially assessed using conventional laboratory testing [amplification refractory mutation system (ARMS) for detecting *EGFR*, *KRAS*, and *BRAF* mutations and immunohistochemistry (IHC) for *ALK* fusions] followed by DNA NGS. Cases negative for driver alterations were further analyzed using RNA NGS to characterize driver alterations in IMA, NMA and mixed IMA/NMA (Fig. 2a). Distinct molecular profiles were observed among the three groups (Fig. 2b). In IMA cases, ARMS and IHC detected driver alterations in 63.0% (220/349), predominantly *KRAS* mutations (46.4%) and *ALK* fusions (13.8%). DNA NGS identified additional driver alterations in 13.5% (47/349) of IMA cases, primarily involving *HER2* mutations (3.7%), *RET* fusions (3.2%), and *NRG1* fusions (3.2%). RNA NGS further revealed driver alterations in 1.7% (6/349) of IMA cases, including *FGFR2* fusions (*n* = 4), *NRG1* fusions (*n* = 1), and *ROS1* fusions (*n* = 1). In mixed IMA/NMA cases, ARMS and IHC detected driver alterations in 70.5% (43/61), most commonly *KRAS* mutations (24.6%), *ALK* fusions (23.0%), and *EGFR* mutations (21.3%). DNA NGS identified additional alterations in 8.2% (5/61) of cases, and RNA NGS detected one additional alteration (1.6%, 1/61), specifically an *SQSTM1-NTRK3* fusion. In NMA cases, ARMS and IHC identified driver alterations in 79.0% (259/328), predominantly *EGFR* mutations (67.7%). DNA NGS revealed additional driver alterations in 11.9% (39/328) of NMA cases, and RNA NGS identified one further alteration (0.3%, 1/328), namely a *CD74-ROS1* fusion (Fig. 2b). Overall, driver alterations were identified in 91.2% of NMA cases, significantly higher than in IMA (78.2%) and mixed IMA/NMA (80.3%, *P* < 0.001) (Fig. 2c). Notably, *EGFR* mutations were detected by DNA NGS but not by ARMS in 15 cases, primarily involving *EGFR* exon 20 insertions (Table S1). In terms of variant types, no *MET* amplification was detected in these cases. A higher proportion of gene fusions (*ALK*, *ROS1*, *RET*, *NRG1*, *NTRK* and *FGFR2*) was observed in both IMA (24.4%) and mixed IMA/NMA (29.5%) groups compared to the NMA group (6.7%, *P* < 0.001). However, mutations *(EGFR*, *KRAS*, *BRAF*, *HER2*, and *MET* exon 14 skipping) were more frequently identified in the NMA group (84.5%) than in IMA (53.9%) and mixed IMA/NMA (50.8%) groups (*P* < 0.001; Fig. 2d). Specifically, *EGFR* mutations exhibited a gradient distribution, with the highest incidence in the NMA group (68.6%, 225/328), significantly higher than in the mixed IMA/NMA (26.2%, 16/61) and IMA groups (1.7%, 6/349; *P* < 0.001; Fig. 2e). Conversely, *KRAS* mutations showed an inverse pattern, with the highest frequency in the IMA group (59.0%, 206/349), followed by the mixed IMA/NMA (29.5%, 18/61) and NMA groups (7.0%, 23/328; *P* < 0.001; Fig. 2e). Further analysis revealed that the incidence of *KRAS* G12C mutation was similar between the mixed IMA/NMA (9.8%, 6/61) and IMA groups (13.5%, 47/349; *P* = 0.538), but higher than in the NMA group (2.1%, 7/328; *P* = 0.008; Fig. S1a). In contrast, *KRAS* G12D mutation incidence was significantly lower in mixed IMA/NMA (3.3%, 2/61) and NMA groups (0.9%, 3/328; *P* = 0.177) compared to the IMA group (14.3%, 50/349; *P* < 0.001; Fig. S1b). Notably, *ALK* fusions were more prevalent in the mixed IMA/NMA group (24.6%, 15/61) compared to the IMA (15.5%, 54/349; *P* = 0.094) and NMA groups (2.7%, 9/328; *P* < 0.001; Fig. 2e). No statistical differences were observed in other driver alterations among the three groups, likely due to their low incidence rates. Additionally, HER2, PD-L1, and c-MET protein expression levels were assessed by IHC in all cases. No significant differences in c-MET or HER2 expression were found among the mixed IMA/NMA, IMA and NMA groups (Fig. S1c and S1d). However, the mixed IMA/NMA group had the highest proportion of patients with tumor cell (TC) ≥ 1% compared to the other two groups (Fig. 2f).

### Genomic alterations of different components in mixed IMA/NMA

**Fig. 3:**
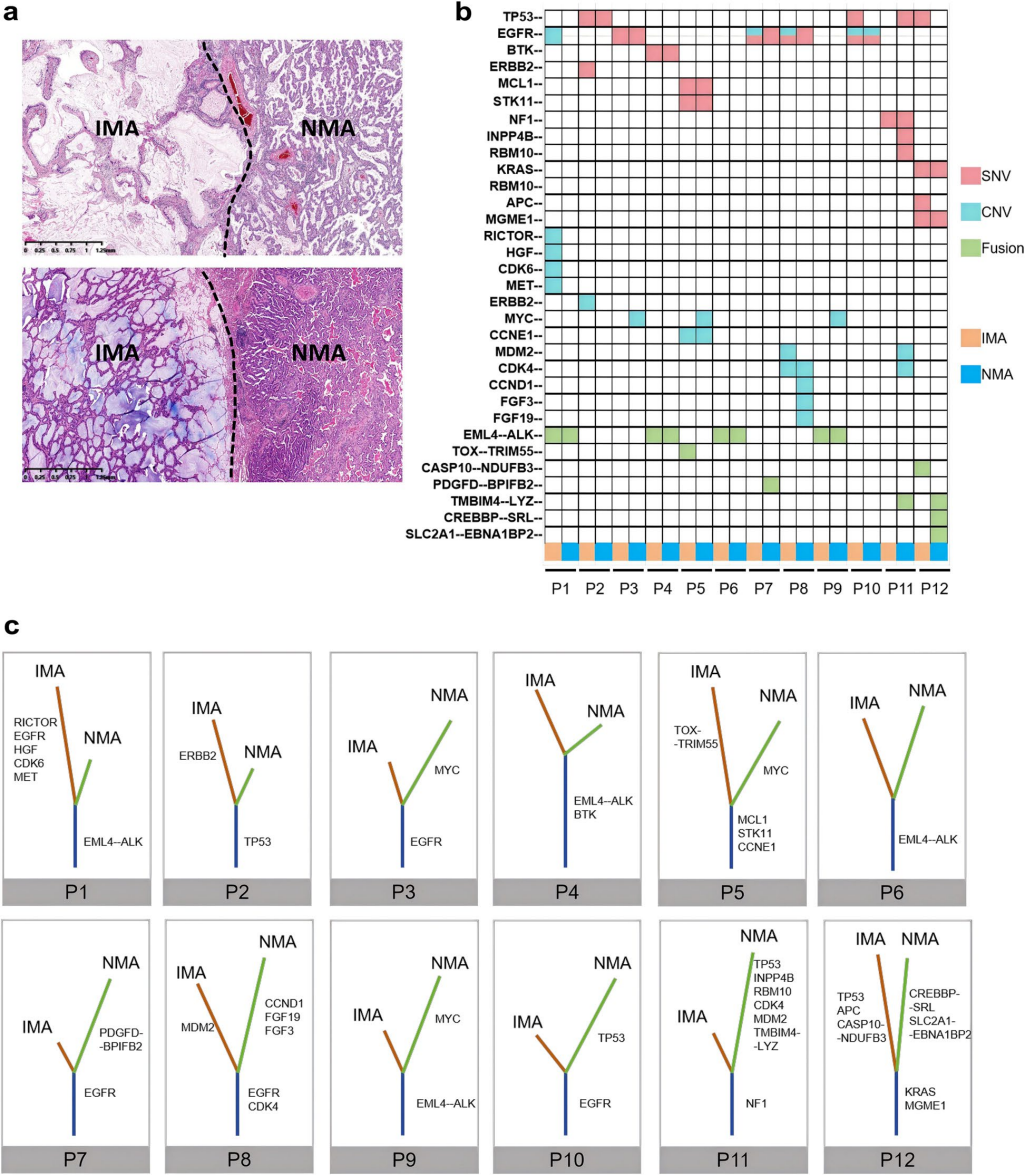
Genetic profiles of IMA and NMA components within the same mixed IMA/NMA case. **a** Schematic illustration of microdissection for IMA and NMA components in mixed IMA/NMA. **b** Data grids showing molecular profiling of the IMA and NMA components. **c** Phylogenetic tree analysis depicting genetic alterations in the IMA and NMA components

To investigate the clonal relationship between distinct histological components (IMA and NMA components) in mixed IMA/NMA, we selected 12 patients with mixed IMA/NMA and performed microdissection to isolate the IMA and NMA components from each tumor (Table S2, Fig. 3a). DNA NGS was performed on samples from each component. Mutations, fusions and copy number variants (CNVs) were analyzed within the same patients. The results revealed 1–3 shared genetic alterations between the IMA and NMA components within individual patients (Fig. 3b). Phylogenetic tree analysis indicated that these shared alterations were predominantly driver genetic events (9/12, 75%), such as *EGFR* mutations, *ALK* fusions or *KRAS* mutations (Fig. 3c). These findings strongly support a common clonal origin of the IMA and NMA components in patients with mixed IMA/NMA.

### Tumor immune microenvironments of different components in mixed IMA/NMA

**Fig. 4:**
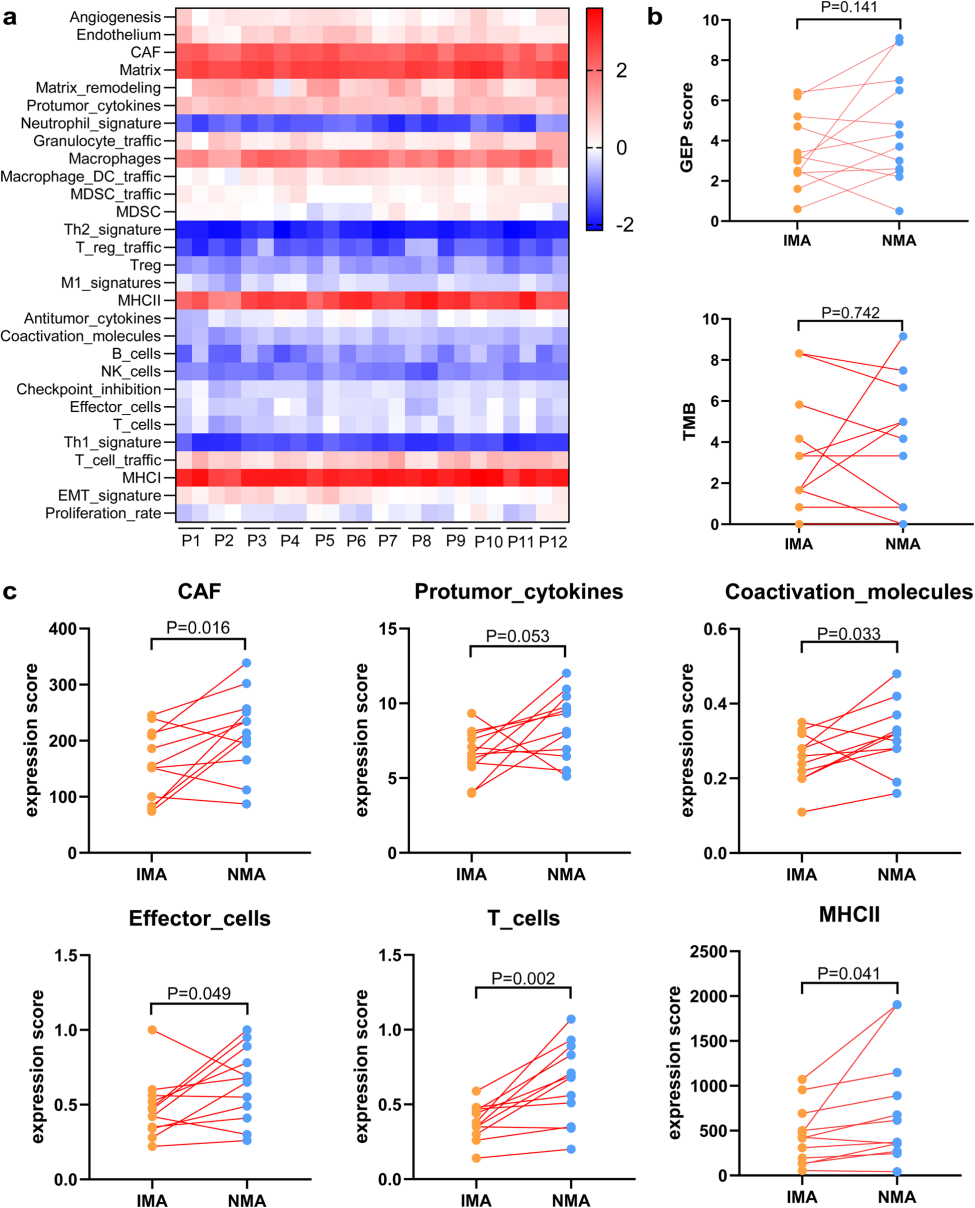
Differences in the tumor microenvironment between IMA and NMA components within the same mixed IMA/NMA case. **a** Heatmap analysis of tumor microenvironment-associated cells and factors in IMA and NMA components. **b** Paired comparison of GEP scores and TMB between IMA and NMA components. **c** Paired comparison of CAF, protumor cytokines, coactivation molecules, effector cells, T cells, and MHC-Ⅱ expression in IMA and NMA components

**Fig. 5:**
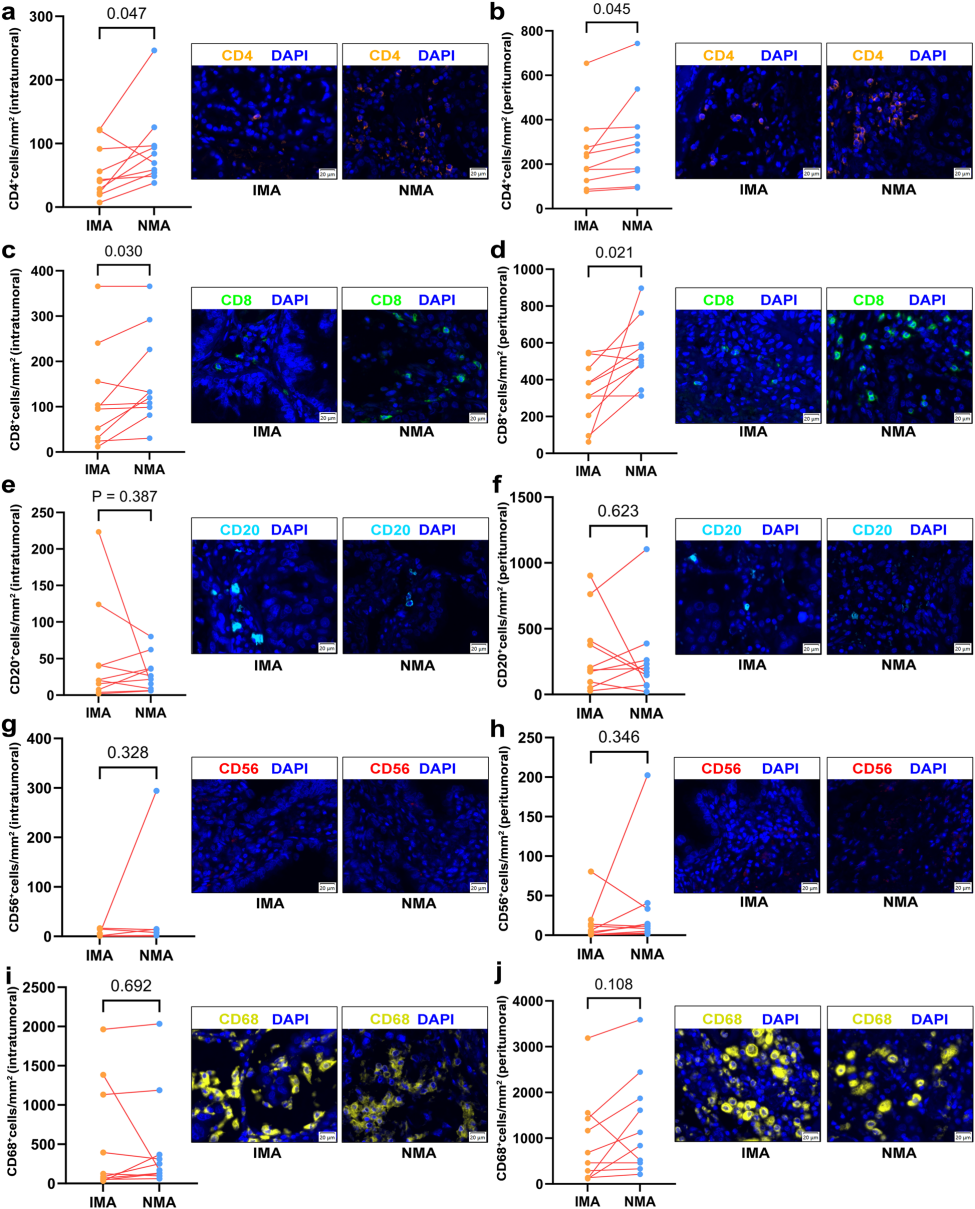
Differences immune cell infiltration between IMA and NMA components in the same mixed IMA/NMA. **a** Paired comparison of intratumoral CD4^+^ cell density in IMA and NMA components. **b** Paired comparison of peritumoral CD4^+^ cell density in IMA and NMA components. **c** Paired comparison of intratumoral CD8^+^ cell density in IMA and NMA components. **d** Paired comparison of peritumoral CD8^+^ cell density in IMA and NMA components. **e** Paired comparison of intratumoral CD20^+^ cell density in IMA and NMA components. **f** Paired comparison of peritumoral CD20^+^ cell density in IMA and NMA components. **g** Paired comparison of intratumoral CD56^+^ cell density in IMA and NMA components. **h** Paired comparison of peritumoral CD56^+^ cell density in IMA and NMA components. **i** Paired comparison of intratumoral CD68^+^ cell density in IMA and NMA components. **j** Paired comparison of peritumoral CD68^+^ cell density in IMA and NMA components

RNA expression of immune-related genes in different components was analyzed using RNA NGS to compare TIME differences between IMA and NMA components within mixed IMA/NMA (*n* = 12, Fig. 4a). No significant differences were observed in T cell-inflamed gene expression profile (GEP) scores or tumor mutation burden (TMB) between the two components (Fig. 4b). However, levels of cancer-associated fibroblasts (CAFs), protumor cytokines, coactivation molecules and MHC-Ⅱ were higher in NMA components compared to IMA components. Moreover, the levels of effector cells and T cells were significantly elevated in NMA components relative to IMA components (Fig. 4c). No significant differences in other TIME-related factors were detected between NMA and IMA components (Fig. S2). In The Cancer Genome Atlas (TCGA) cohort, TIME differences between pure IMA and pure NMA were assessed based on immune cell biomarker expression. The results showed that CD8^+^ T cells, monocytes, M1 macrophages, and dendritic cells were significantly more abundant in NMA cases than in IMA cases (Fig. S3), further supporting distinct TIME profiles between NMA and IMA. To validate these findings, multiplex immunofluorescence (mIF) assays were performed on IMA and NMA components within the same mixed IMA/NMA tumors (*n* = 10). The results demonstrated that higher densities of CD4^+^ and CD8^+^ T cells were observed in NMA components compared to IMA components, both in intratumoral and peritumoral regions (Fig. 5a-d). However, no statistically significant differences were observed between NMA and IMA components in the expression levels of CD20^+^, CD56^+^, or CD68^+^ cells, either within the tumor or in peritumoral areas (Fig. 5e-j). These findings indicate that IMA and NMA components within the same mixed IMA/NMA tumor may harbor distinct tumor immune microenvironments.

### Survival analysis of mixed IMA/NMA

**Fig. 6:**
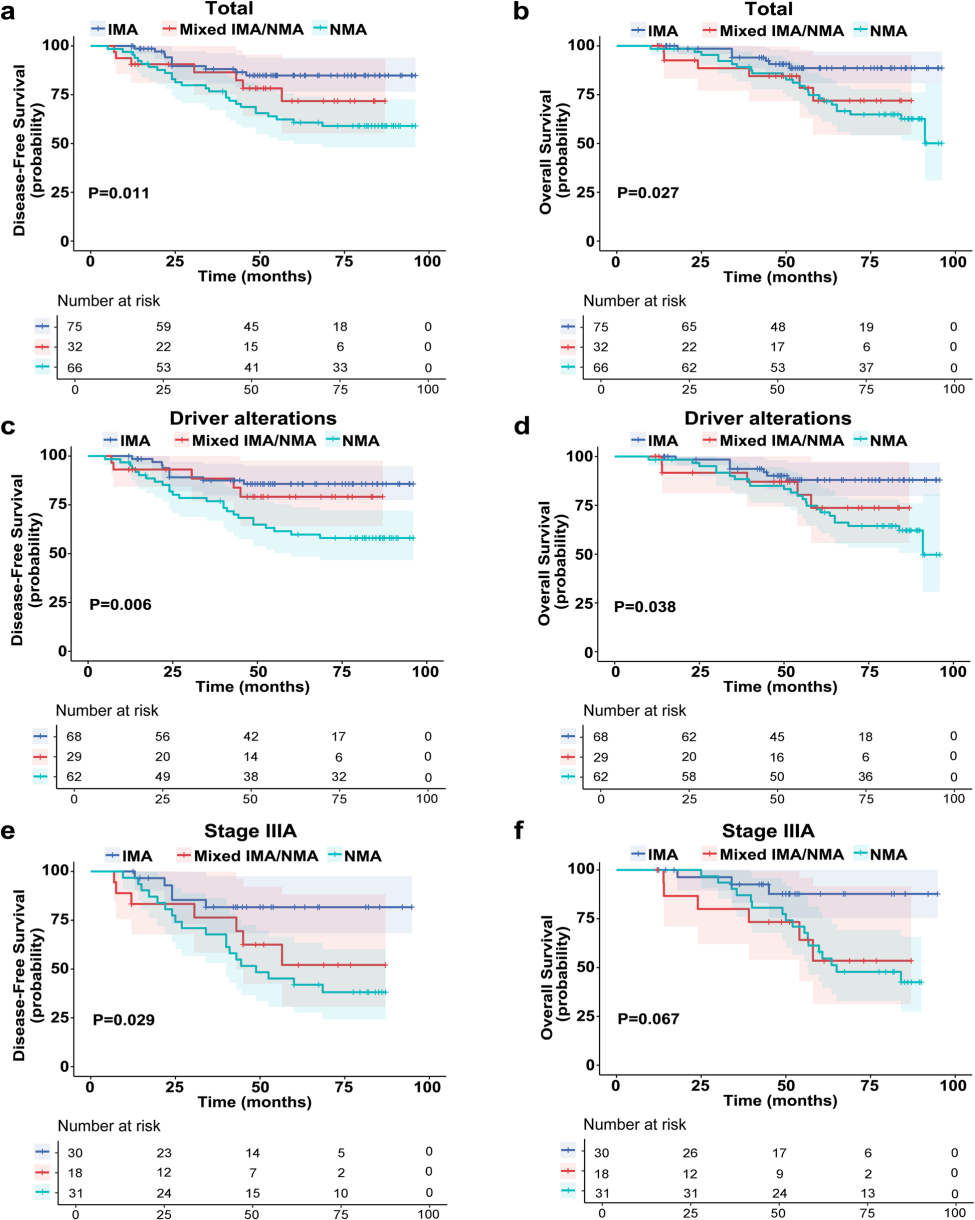
Survival outcomes in patients with different LUAD subtypes. **a** DFS curves for patients with IMA, mixed IMA/NMA and NMA. **b** OS curves for patients with IMA, mixed IMA/NMA and NMA. **c** DFS curves for patients with driver alterations across the three subtypes. **d** OS curves for patients with driver alterations across the three subtypes. **e** DFS curves for patients with stage IIIA disease across the three subtypes. **f** OS curves for patients with stage IIIA disease across the three subtypes

Surgical outcomes were evaluated in 173 patients, including 75 with pure IMA, 32 with mixed IMA/NMA, and 66 with pure NMA. No significant differences in baseline clinicopathological characteristics were observed among the three groups (Table S3). The five-year disease-free survival (DFS) and overall survival (OS) rates were 84.9% and 88.6% in the IMA group, 71.7% and 72.0% in the mixed IMA/NMA group, and 60.7% and 73.2% in the NMA group, respectively (*P* = 0.011 for DFS; *P* = 0.027 for OS) (Fig. 6a and b). Among patients with driver alterations, the trend persisted: the IMA group exhibited significantly better five-year DFS (85.7%, *P* = 0.006) and OS (88.0%, *P* = 0.038) compared to the mixed IMA/NMA group (DFS 79.1%, OS 73.7%) and the NMA group (DFS 59.8%, OS 71.4%) (Fig. 6c and d). In subgroup analysis stratified by TNM stage, stage ⅢA patients in the IMA group showed improved five-year DFS (81.7%, *P* = 0.029) and OS (87.7%, *P* = 0.067) compared to those in the mixed IMA/NMA (DFS 52.1%, OS 53.5%) and NMA (DFS 41.9%, OS 54.6%) groups (Fig. 6e and f), although the OS difference did not reach statistical significance. Univariate and multivariate analyses of key variables, including age, sex, smoking history, driver alterations, stage, and pathological subtype, revealed that stage and pathological subtypes were the independent prognostic factors for both DFS and OS (Table 2). Both mixed IMA/NMA and NMA were significantly associated with poorer survival outcomes.

**Table 2:** Univariate and multivariate analyses of factors associated with DFS and OS

Variable	DFS				OS			
	Univariable Analysis	P	Multivariable Analysis	P	Univariable Analysis	P	Multivariable Analysis	P
	HR (95% CI)		HR (95% CI)		HR (95% CI)		HR (95% CI)	
Age (< 60 vs. ≥ 60 y)	0.57 (0.27–1.18)	0.127	-		1.81 (0.80–3.06)	0.156	-	
Sex (male vs. female)	1.30 (0.96–1.74)	0.088	-		1.33 (0.97–1.83)	0.148	-	
Smoking history (yes vs. no)	1.03 (0.85–1.18)	0.116	-		1.03 (0.85–1.18)	0.121	-	
Driver alterations (yes vs. no)	0.87 (0.37–2.05)	0.745	0.97 (0.40–2.35)	0.941	1.06 (0.63–1.79)	0.816	1.29 (0.44–3.77)	0.693
Stage (I/II vs. IIIA)	0.25 (0.13–0.49)	< 0.001	0.27 (0.14–0.53)	< 0.001	0.30 (0.15–0.60)	< 0.001	0.32 (0.16–0.65)	0.001
Pathological subtypes (compared with IMA)								
NMA	2.89 (1.39–5.99)	0.004	2.64 (1.26–5.52)	0.010	3.01 (1.29–7.01)	0.011	2.69 (1.15–6.33)	0.023
Mixed IMA/NMA	2.47 (1.01–6.09)	0.049	2.04 (0.82–5.05)	0.034	3.30 (1.20–9.14)	0.021	2.85 (1.02–7.97)	0.045

*HR* hazard ratio, *DFS* disease-free survival, *OS* overall survival

## Discussion

In this study, we demonstrated that mixed IMA/NMA exhibited distinct molecular features compared to IMA and NMA, characterized by a unique distribution of driver alterations, particularly in *EGFR*, *KRAS*, and *ALK*. Furthermore, intratumoral comparison revealed shared genetic alterations between the IMA and NMA components within the same mixed IMA/NMA cases, suggesting a monoclonal origin. However, heterogeneous tumor microenvironments were observed across different components of the same mixed IMA/NMA tumors, which may contribute to differential clinical responses to immunotherapy. Clinically, mixed IMA/NMA demonstrated a survival pattern similar to that of NMA, but with significantly shorter DFS and OS compared to IMA. These findings indicate that mixed IMA/NMA represents a biologically and prognostically distinct subtype.

It has been reported that *KRAS* mutation is the most common driver alteration in IMA [11], whereas NMA is characterized by a high *EGFR* mutation rate. However, the molecular profile of mixed IMA/NMA remains unclear due to the rare incidence. In this study, through analysis of a large cohort involving NMA, IMA and mixed IMA/NMA, we found that mixed IMA/NMA exhibited unique intermediate molecular features with asymmetric characteristics. The *KRAS* mutation rate in mixed IMA/NMA was lower than that in IMA but higher than that in NMA, while the *EGFR* mutation rate in mixed IMA/NMA was lower than that in NMA but higher than that in IMA. To comprehensively characterize the molecular profile of mixed IMA/NMA, sequential multi-platform molecular testing, including ARMS, IHC, DNA NGS and RNA NGS were performed. DNA NGS is widely used in clinical practice due to its broader mutation detection capability compared to conventional laboratory methods such as ARMS. Consequently, several *EGFR* mutations were identified by DNA NGS in ARMS-negative cases, particularly in the NMA and mixed IMA/NMA groups. Furthermore, RNA NGS enhances the detection of fusions, and the combined use of DNA and RNA NGS has been shown to improve the identification of diverse genomic alterations in LUAD [14, 15]. However, the optimal strategy-sequential versus parallel DNA and RNA sequencing-remains controversial for LUAD [16]. In our cohort, the fusion rate was markedly higher in mixed IMA/NMA and IMA compared to NMA, supporting the notion that gene fusions are frequently associated with mucin production in LUAD [17]. Additionally, RNA NGS detected a higher proportion of fusions missed by DNA NGS in mixed IMA/NMA and IMA, including *ROS1*, *NRG1*, *NTRK* and *FGFR2* fusions, primarily due to the limitation of short-read sequencing technologies [18]. Given the distinct driver landscapes and mutational spectra across histological subtypes, optimized molecular testing strategies are warranted. The increased detection yield of RNA NGS in mixed IMA/NMA and IMA suggests that comprehensive molecular profiling, such as parallel DNA and RNA sequencing, may be beneficial for these LUAD subtypes. PD-L1 expression has been established as a biomarker associated with response to immune checkpoint inhibitors in LUAD [19]. Currently, only a limited number of studies have investigated PD-L1 expression in IMA [20, 21], consistently reporting lower PD-L1 levels in IMA compared to NMA [17]. In this study, our results showed a higher proportion of patients with PD-L1 expression ≥ 1% in mixed IMA/NMA than either pure IMA or NMA, suggesting a more immunoinflammatory tumor microenvironment in the mixed subtype [22, 23].

To date, no studies have reported on the clonal relationship or tumor heterogeneity in mixed IMA/NMA. Our DNA NGS analyses revealed 1–3 shared somatic alterations between the IMA and NMA components within the same tumor, indicating a common clonal origin. Despite this shared ancestry, each component harbored distinct private mutations, supporting the model of intratumoral heterogeneity driven by genomic instability and microenvironmental selection pressures [24, 25]. As most shared alterations were driver mutations, targeted therapies may theoretically be effective against both components; however, private mutations may contribute to differential treatment responses and eventual therapeutic resistance. Further studies are needed to evaluate clinical responses to targeted therapies across different histological subtypes (IMA, NMA and mixed IMA/NMA). Additionally, RNA NGS and mIF were performed to compare the TIME between IMA and NMA components in mixed IMA/NMA cases. RNA NGS revealed notable differences in TIME-related factors between components. The NMA components were enriched in CAFs, protumor-cytokines, coactivating molecules, MHC-II, T cells, and effector cells, features associated with active cellular immunity [26]. In contrast, the IMA components displayed an immune-desert phenotype [27]. Transcriptomic analysis of the TCGA cohort further confirmed higher immune activity in NMA compared to IMA. Moreover, mIF analysis demonstrated higher densities of CD4^+^ and CD8^+^ T cells in NMA components relative to IMA components, further validating intratumoral heterogeneity in the immune microenvironment of mixed IMA/NMA.

The incidence of IMA is relatively low, and studies on the prognosis of IMA remain limited, with inconsistent conclusions. Luo et al. and Cui et al. reported no significant difference in overall survival OS or DFS between IMA and NMA [28, 29]. In contrast, Xu et al. and Gow et al. found a more favorable prognosis for IMA compared to NMA [30, 31]. Notably, variations in histological classification criteria and stage stratification may contribute to these inconsistent prognostic findings. In some previous studies, mixed IMA/NMA was not analyzed as an independent subtype but was instead grouped with IMA [28]. By clearly distinguishing pure IMA, pure NMA, and mixed IMA/NMA, our study demonstrated that patients with IMA had significantly better survival outcomes than those with NMA following surgery. However, patients with mixed IMA/NMA exhibited survival outcomes comparable to those with NMA, with significantly worse DFS and OS compared to patients with IMA. This survival trend persisted after stratification by driver gene alteration status and adjuvant chemotherapy (stage IIIA). Both NMA and mixed IMA/NMA subtypes were associated with poorer surgical outcomes, suggesting that the presence of the NMA component may contribute to more aggressive biological behavior [32, 33].

This study has several limitations. First, due to the extremely low incidence of mixed IMA/NMA, the sample size of the mixed IMA/NMA group was smaller compared to the pure IMA and NMA groups. Second, only 12 mixed IMA/NMA cases were available for further comparative analysis of the IMA and NMA components, limiting the exploration of clonal origin and tumor microenvironment characteristics. Third, the absence of stage IV cases precluded evaluation of survival outcomes in advanced disease.

In summary, this study revealed that mixed IMA/NMA exhibited distinct molecular and clinical characteristics compared to pure IMA and NMA, particularly in terms of sex, smoking history, clinical stage, *EGFR*/*KRAS*/*ALK* alterations and PD-L1 expression. Furthermore, we comprehensively characterized the clonal relationship and heterogeneity in mixed IMA/NMA, identifying clonal relatedness in the genomic landscape but distinct heterogeneity in the immune microenvironment between the IMA and NMA components within the same patient. Additionally, both mixed IMA/NMA and NMA were associated with more aggressive clinical behavior than IMA, with shorter DFS and OS following surgery. These findings provide valuable insights for future research on mixed IMA/NMA, and offer important implications for the precise diagnosis and management of this rare LUAD subtype.

## Materials and methods

### Patients

A total of 738 patients with LUAD treated at the Cancer Hospital, Chinese Academy of Medical Sciences and Peking Union Medical College between January 2019 and September 2024 were enrolled. Inclusion criteria were: (i) age ≥ 18 years; (ii) completion of surgical resection; (iii) pathologically confirmed diagnosis of mixed IMA/NMA, pure NMA, or pure IMA; and (iv) availability of sufficient tumor tissue for histological and molecular analyses. Exclusion criteria were: (i) tumor samples with less than 20% tumor cellularity; or (ii) receipt of neoadjuvant therapy. This study was conducted in accordance with the principles of the Declaration of Helsinki. Approval was granted by the Ethics Committee of the National Cancer Center/National Clinical Research Center for Cancer/Cancer Hospital, Chinese Academy of Medical Sciences and Peking Union Medical College (NCC-007943). Informed consent was obtained from all individual participants included in the study.

### Clinical parameters and histopathological evaluation

All clinical data, including age, sex, clinical stage, and smoking history, were extracted from medical records. Following surgical resection, all samples were fixed in formalin, embedded in paraffin, and stained with hematoxylin and eosin (HE). Pathological diagnoses were independently made by two experienced pathologists. Mixed IMA/NMA was diagnosed when the tumor consisted of both mucinous and non-mucinous components, with each component comprising at least 10% of the tumor [13].

### IHC

IHC was performed to evaluate the protein expression of ALK, HER2, c-MET, and PD-L1 in tumor tissues. Briefly, tumor tissue sections were stained using an autostainer (Autostainer Link 48, Dako, Denmark) with primary antibodies against ALK (Roche, Basel, Switzerland), HER2 (Roche, Basel, Switzerland), c-MET (Roche, Basel, Switzerland), and PD-L1 (SP263, Ventana Medical Systems Inc, Tucson, USA). For PD-L1 expression, the TC score was defined as the percentage of viable tumor cells showing partial or complete membrane staining for PD-L1 at any intensity, relative to all viable tumor cells in the sample. PD-L1 expression was considered negative when TC < 1% and positive when TC ≥ 1%. For HER2 and c-MET expressions, IHC staining intensity was graded as 0 (no staining), 1 + (weak), 2 + (moderate), or 3 + (strong). Tumors with a staining intensity of 2+ or 3 + were recorded as positive, and those with 0 or 1 + as negative. For ALK expression, ALK positive was identified as previously reported [34]. All results were independently assessed by two experienced pathologists.

### ARMS

Genomic DNA was extracted from tumor tissue using the Concertbio DNA FFPE Tissue Kits (AmoyDx, Fujian, China) according to the manufacturer’s instructions. Human *KRAS* mutation detection kit (Accbio, Beijing, China), human *EGFR* mutation detection kit (Accbio, Beijing, China) and human *BRAF* mutation detection kit (Accbio, Beijing, China) were used to detect mutations of *EGFR*, *KRAS*, and *BRAF,* respectively (Table S4). Real-time PCR was performed as previously described [35, 36]: an initial denaturation at 95 °C for 5 min, followed by 40 cycles of 95 ℃ for 15 s, and 60 ℃ for 1 min. Mutations were determined according to threshold count values, following the manufacturer's instructions.

### Hybridization capture‑based targeted NGS

DNA and RNA were extracted separately from tissue samples. Pre-libraries were constructed through ultrasonic fragmentation, end repair, and PCR amplification. Hybridization capture was performed on the pre-libraries using a 571-gene panel for DNA NGS (Table S5) and/or a 2660-gene panel for RNA NGS (Table S6), followed by PCR amplification and purification to generate the final libraries. Sequencing was carried out on the Illumina platform. DNA NGS was used to identify mutations, gene fusions, amplifications and TMB (a potential predictive biomarker for the efficacy of immune checkpoint inhibitors defined as the total number of somatic mutations per million bases sequenced in the tumor genome), while RNA NGS was employed to assess the expression of genes associated with TIME. The T cell-inflamed GEP score, a specific gene expression signature reflecting the immune status within the tumor microenvironment, was computed as the weighted sum of normalized expression values of 18 genes (*TIGIT*, *CD27*, *CD8A*, *PDCD1LG2*, *LAG3*, *CD274*, *CXCR6*, *CMKLR1*, *NKG7*, *CCL5*, *PSMB10*, *IDO1*, *CXCL9*, *HLA-DQA1*, *CD276*, *STAT1*, *HLA-DRB1*, and *HLA-E*) [37]. Similarly, other TIME-related factors, including protumor cytokines (*IL10*, *TGFB1*, *TGFB2*, *TGFB3*, *IL22*, *MIF*, and *IL6*), coactivation molecules (*CD28*, *CD40*, *TNFRSF4*, *ICOS*, *TNFRSF9*, *CD27*, *CD80*, *CD86*, *CD40LG*, *CD83*, *TNFSF4*, *ICOSLG*, *TNFSF9*, and *CD70*), and effector cells (*IFNG*, *GZMA*, *GZMB*, *PRF1*, *GZMK*, *ZAP70*, *GNLY*, *FASLG*, *TBX21*, *EOMES*, *CD8A*, and *CD8B*), were also quantified using the weighted sum of their normalized expression values.

### mIF

Multiplex immunofluorescence was performed to characterize immune cell subsets within the TIME. The PANO 7-plex IHC Kit (Panovue, Beijing, China) was used to detect PanCK, CD4, CD8, CD20, CD56 and CD68. Primary antibodies were applied sequentially, followed by incubation with horseradish peroxidase-conjugated secondary antibodies and tyramide signal amplification (TSA). After labeling all target antigens, nuclei were counterstained with DAPI. Whole-slide fluorescence images were acquired using an Olympus VS200 scanner (Olympus, Germany) equipped with UPLXAPO 20 × objective lens and analyzed using QuPath software. PanCK was used to delineate tumor regions, and cell densities in intratumoral and peritumoral areas were calculated, separately.

### Patient follow-up

Surgical outcomes were evaluated on the basis of survival endpoints, including DFS and OS. All patients with stage IIIA received adjuvant chemotherapy postoperatively. Patient survival status was obtained through medical records or telephone interviews. The follow-up period spanned from the first postoperative day to February 15, 2026, with median follow-up of 60.0 months (95% confidence interval [CI] 55.7–63.0 months). DFS was defined as the time from surgery to the first occurrence of local recurrence or distant metastasis; OS was defined as the time from surgery to death from any cause.

### The TCGA cohort

Clinical and transcriptomic data for NMA (*n* = 67) and IMA (*n* = 16) were retrieved from TCGA via cBioPortal. Data usage followed the TCGA publication guidelines and the NIH Genomic Data User Code of Conduct. No cases of mixed IMA/NMA subtype were identified in the TCGA database, likely due to its rarity.

### Statistical analysis

Statistical analyses were performed using SPSS 25.0 software (IBM, Chicago, IL). Clinical data, including sex, age, clinical stage, and smoking history were compared using the Chi-square test. Mutations in tumor tissues across the three groups were detected and compared using the Chi-square test. The GEP scores, TMB, and expression levels of immunological markers in the mucinous and non-mucinous components of the same mixed IMA/NMA were evaluated using the paired t-test. Univariate and multivariate analyses were conducted using the Cox proportional hazards regression model. Statistical significance was defined as a two-sided *P* value of < 0.05.

## Supplementary Information

Supplementary Material 1.

## Data Availability

All data and materials supporting the findings of this study are available from the corresponding author upon reasonable request. TCGA data used in this study were obtained from the cBioPortal. All data were downloaded on March 26, 2025, and were used in accordance with applicable data use policies.
