# Supplementary material for: Molecular characteristics, clonal relatedness and surgical outcomes of pulmonary mixed invasive mucinous and non-mucinous adenocarcinoma: a retrospective cohort study
Source: Mol Biomed. 2026 May 9;7:65. doi: 10.1186/s43556-026-00460-1 (PMC13157374; doi:10.1186/s43556-026-00460-1)
Supplement: Supplementary file 1 — Supplementary Material 1. [file 43556_2026_460_MOESM1_ESM.docx]

**Molecular characteristics,** **clonal relatedness and** **surgical outcomes** **of pulmonary mixed invasive mucinous and non-mucinous adenocarcinoma: a retrospective cohort study**

**Running title:** Characterization and profiling of pulmonary mixed IMA/NMA

Xinyi Shi^1,2,#^, Yang Wang^3,#^, Nan Yao^1,#^, Bowen Xue^1,#^, Lei Guo^1,#^, Liming Xu^4^, Changbin Zhu^4^, Guiping Qin^2,*^ Jianming Ying^1,*^, Yutao Liu^3,*^, Weihua Li^1,*^

^1^Department of Pathology, State Key Laboratory of Molecular Oncology, National Cancer Center/National Clinical Research Center for Cancer/Cancer Hospital, Chinese Academy of Medical Sciences and Peking Union Medical College, Beijing, China

^2^Department of Pathology, Liangxiang Hospital of Beijing Fangshan District, Beijing, China

^3^Department of Medical Oncology, National Cancer Center/National Clinical Research Center for Cancer/Cancer Hospital, Chinese Academy of Medical Sciences and Peking Union Medical College, Beijing, China;

^4^Amoy Diagnostics Co., Ltd., Xiamen, China

^#^ Xinyi Shi, Yang Wang, Nan Yao, Bowen Xue and Lei Guo contributed equally to this work.

*Corresponding author:

Dr. Weihua Li, Dr. Yutao Liu, Dr. Jianming Ying, and Dr. Guiping Qin

E-mail: liweihua@cicams.ac.cn (Li W.); [liuyutao@cicams.ac.cn](mailto:liuyutao@cicams.ac.cn) (Liu Y.); jmying@cicams.ac.cn (Ying J.); and 1164125929@qq.com (Qin G.).


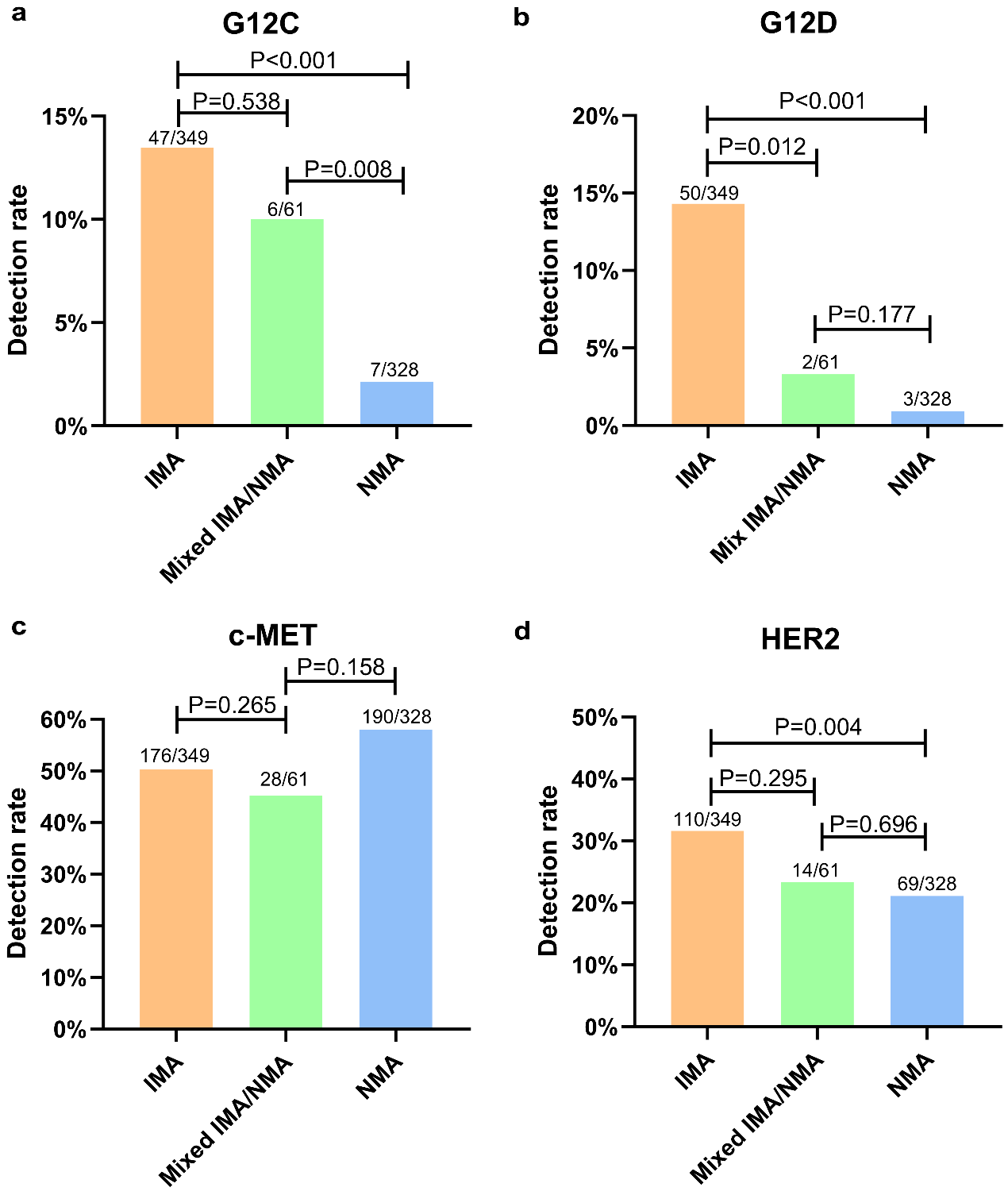


**Fig. S1** Molecular phenotypic differences among IMA, NMA, and mixed IMA/NMA groups. (a) Detection rates of *KRAS* G12C mutation in IMA, NMA, and mixed IMA/NMA groups. (b) Detection rates of *KRAS* G12D mutation in IMA, NMA, and mixed IMA/NMA groups. (c) Expression levels of c-MET in IMA, NMA, and mixed IMA/NMA groups. (d) Expression levels of HER2 in IMA, NMA, and mixed IMA/NMA groups.


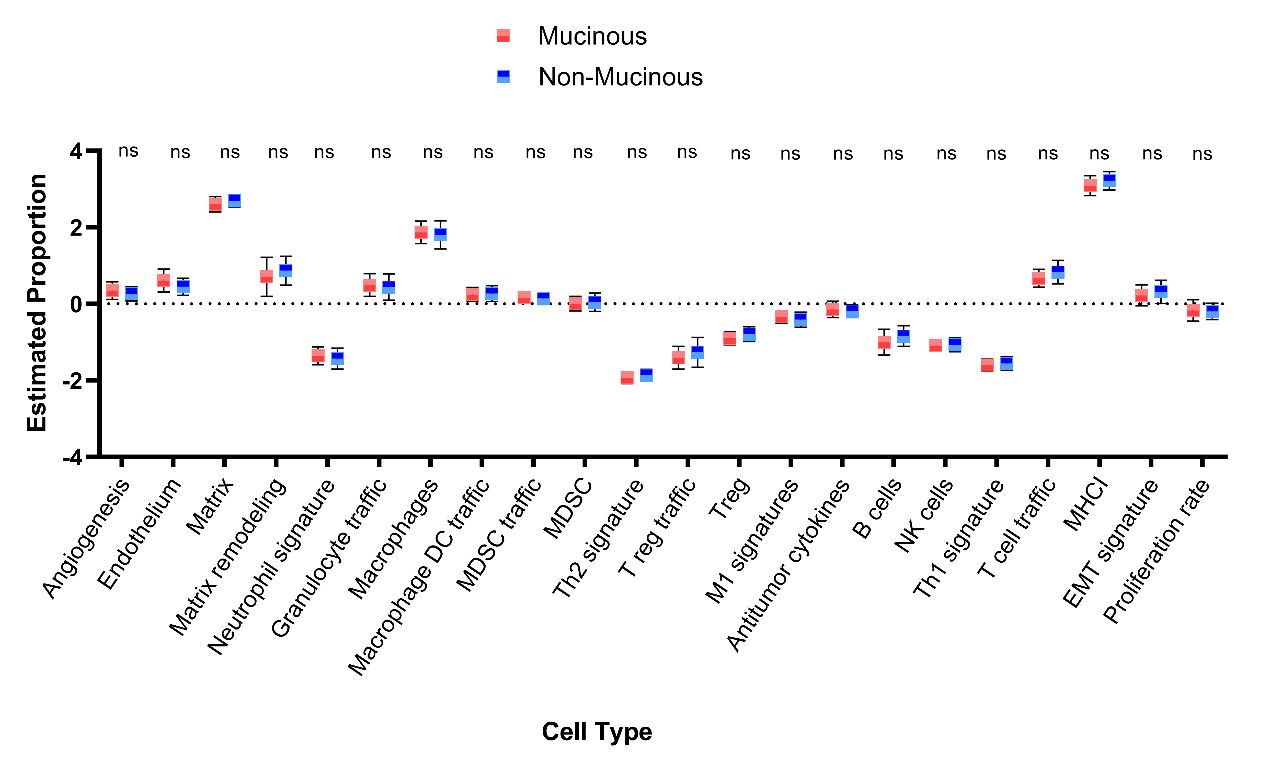


**Fig. S2** Levels of tumor microenvironment-related cells and factors between IMA and NMA components in mixed IMA/NMA cases.


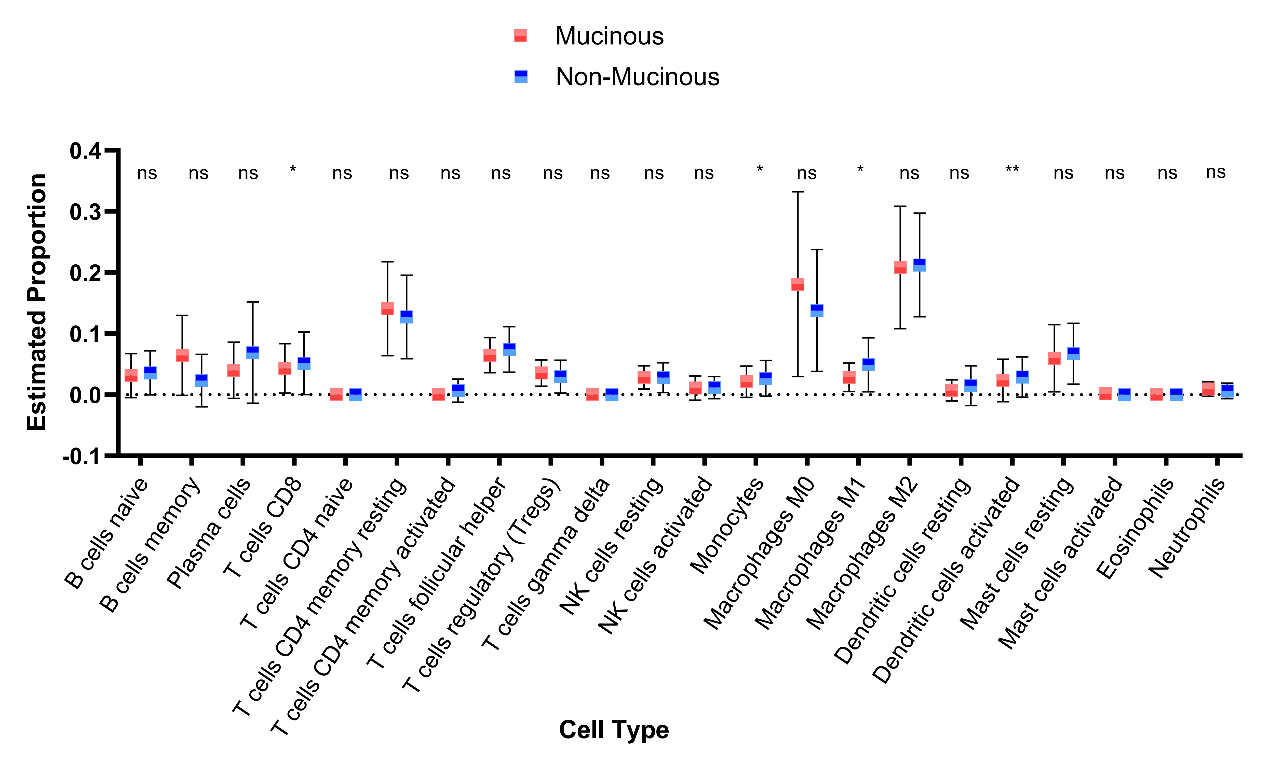


**Fig. S3** Immune cell composition in the tumor microenvironment of pure IMA and NMA cases from the TCGA database.

| **Table S1** Driver alterations not detected by ARMS or DNA NGS. | | | | |
| --- | --- | --- | --- | --- |
| **Patient** | **Gene** | **Exon** | **Alteration** | **Mutation allele fraction** |
| ***EGFR* mutations detected by DNA NGS but not ARMS** | | | | |
| P512 | *EGFR* | 21 | p.L861R | 31.0% |
| P534 | *EGFR* | 20 | p.H773_V774insAH | 16.2% |
| P547 | *EGFR* | 20 | p.H773_V774insAH | 66.1% |
| P598 | *EGFR* | 20 | p.N771>KG | 42.5% |
| P611 | *EGFR* | 19 | p.E709_T710>D | 16.5% |
| P631 | *EGFR* | 20 | p.P772_H773dup | 8.8% |
| P652 | *EGFR* | 18 | p.E709_T710>D | 85.5% |
| P689 | *EGFR* | 19 | p.T751_I759>N | 7.6% |
| P699 | *EGFR* | 18 | p.E709_T710>D | 24.0% |
| P702 | *EGFR* | 20 | p.N771_P772insH | 8.4% |
| P703 | *EGFR* | 20 | p.N771>KH | 11.6% |
| P711 | *EGFR* | 18 | p.E709_T710>D | 19.5% |
| P723 | *EGFR* | 20 | p.N771delinsGTH | 13.7% |
| P728 | *EGFR* | 20 | p.H773>NPY | 8.2% |
| P731 | *EGFR* | 20 | p.A763_Y764insFQEA | 15.2% |
| **Fusions detected by RNA NGS but not DNA NGS** | | | | |
| P21 | *FGFR2* | ex17:ex3 | *FGFR2*-*MBIP* |  |
| P133 | *FGFR2* | ex17:ex2 | *FGFR2*-*BAIAP2L1* |  |
| P189 | *FGFR2* | ex17:ex3 | *FGFR2*-*MAX* |  |
| P277 | *FGFR2* | ex17:ex24 | *FGFR2*-*CIT* |  |
| P301 | *NRG1* | ex2:ex2 | *ATP1B1*-*NRG1* |  |
| P312 | *ROS1* | ex6:ex34 | *CD4*-*ROS1* |  |
| P384 | *NTRK3* | ex4:ex14 | *SQSTM1*-*NTRK3* |  |
| P545 | *ROS1* | ex6:ex34 | *CD4*-*ROS1* |  |

| **Table S2** Clinicopathological characteristic of the 12 patients with mixed IMA/NMA. | | | | | | |
| --- | --- | --- | --- | --- | --- | --- |
| Patient ID | SEX | Age | Tumor ID | Smoking | cTNM | pTNM |
| P1 | Male | 42 | IMA | Never | Ⅲa | pT2aN2 |
|  |  |  | NMA |  |  |  |
| P2 | Male | 64 | IMA | Ex- or current | Ⅲb | pT3N2 |
|  |  |  | NMA |  |  |  |
| P3 | Male | 48 | IMA | Never | Ⅰa | pT1cN0 |
|  |  |  | NMA |  |  |  |
| P4 | Female | 71 | IMA | Never | Ⅲa | pT1bN2 |
|  |  |  | NMA |  |  |  |
| P5 | Male | 63 | IMA | Ex- or current | Ⅲa | pT4N0 |
|  |  |  | NMA |  |  |  |
| P6 | Female | 47 | IMA | Never | Ⅰa | pT1bN0 |
|  |  |  | NMA |  |  |  |
| P7 | Female | 75 | IMA | Never | Ⅲa | pT2bN2 |
|  |  |  | NMA |  |  |  |
| P8 | Female | 45 | IMA | Never | Ⅰb | pT2a(m)N0 |
|  |  |  | NMA |  |  |  |
| P9 | Male | 55 | IMA | Ex- or current | Ⅳa | pT2N2M1a |
|  |  |  | NMA |  |  |  |
| P10 | Female | 67 | IMA | Never | Ⅱb | pT3N0 |
|  |  |  | NMA |  |  |  |
| P11 | Male | 67 | IMA | Never | Ⅰa | pT1bN0 |
|  |  |  | NMA |  |  |  |
| P12 | Male | 58 | IMA | Ex- or current | Ⅲa | pT4N0 |
|  |  |  | NMA |  |  |  |

**Table S3** Baseline characteristics of patients who underwent long-term follow-up.

| **Patient** | **Total** | **IMA** | **Mixed IMA/NMA** | **NMA** | ***P* Value** |
| --- | --- | --- | --- | --- | --- |
| **Age** |  |  |  |  |  |
| ≤60 | 96 (55.5%) | 37 (49.3%) | 18 (56.3%) | 41 (62.1%) | 0.311 |
| >60 | 77 (44.5%) | 38 (50.7%) | 14 (43.7%) | 25 (37.9%) |  |
| **Sex** |  |  |  |  |  |
| Male | 82 (47.4%) | 29 (38.7%) | 20 (62.5%) | 32 (48.5%) | 0.073 |
| Female | 91 (52.6%) | 46 (61.3%) | 12 (37.5%) | 34 (51.5%) |  |
| **Stage** |  |  |  |  |  |
| Ⅰ/Ⅱ | 94 (54.3%) | 45 (60.0%) | 14 (43.7%) | 35 (53.0%) | 0.292 |
| ⅢA | 79 (45.7%) | 30 (40.0%) | 18 (56.3%) | 31 (47.0%) |  |
| **Driver alterations** |  |  |  |  |  |
| Yes | 159 (91.9%) | 68 (90.7%) | 29 (90.6%) | 62 (93.9%) | 0.735 |
| No | 14 (8.1%) | 7 (9.3%) | 3 (9.4%) | 4 (6.1%) |  |
| **Smoking** |  |  |  |  |  |
| Ex- or current | 61 (35.3%) | 21 (28.0%) | 16 (50.0%) | 24 (36.4%) | 0.090 |
| Never | 112 (64.7%) | 54 (72.0%) | 16 (50.0%) | 42 (63.6%) |  |

**Table S4** Mutations in *EGFR*, *KRAS* and *BRAF* detected by ARMS.

| **Gene** | **Exon** | **Base change** | **Amino acid change** |
| --- | --- | --- | --- |
| *EGFR* | 18 | c.2155G>A | p.G719S |
|  | 18 | c.2155G>T | p.G719C |
|  | 18 | c.2156G>C | p.G719A |
|  | 19 | c.2235_2249del15 | p.E746_A750delELREA |
|  | 19 | c.2236_2250del15 | p.E746_A750delELREA |
|  | 19 | c.2237_2251del15 | p.E746_T751>A |
|  | 19 | c.2235_2252>AAT | p.E746_T751>I |
|  | 19 | c.2238_2252del15 | p.L747_T751delLREAT |
|  | 19 | c.2240_2254del15 | p.L747_T751delLREAT |
|  | 19 | c.2233_2247del15 | p.K745_E749delKELRE |
|  | 19 | c.2235_2251>AG | p.E746_T751>A |
|  | 19 | c.2240_2257del18 | p.L747_P753>S |
|  | 19 | c.2239_2256del18 | p.L747_S752delLREATS |
|  | 19 | c.2238_2255del18 | p.E746_S752>D |
|  | 19 | c.2239_2258>C | p.L747_P753>Q |
|  | 19 | c.2236_2253del18 | p.E746_T751delELREAT |
|  | 19 | c.2237_2254del18 | p.E746_S752>A |
|  | 19 | c.2237_2255>T | p.E746_S752>V |
|  | 19 | c.2235_2252del18 | p.E746_T751delELREAT |
|  | 19 | c.2235_2255>AAT | p.E746_S752>I |
|  | 19 | c.2237_2256>TC | p.E746_S752>V |
|  | 19 | c.2238_2248>GC | p.L747_A750>P |
|  | 19 | c.2239_2247delTTAAGAGAA | p.L747_E749delLRE |
|  | 19 | c.2239_2248TTAAGAGAAG>C | p.L747_A750>P |
|  | 19 | c.2236_2248>CAAC | p.E746_A750>QP |
|  | 19 | c.2236_2248>AGAC | p.E746_A750>RP |
|  | 19 | c.2235_2248>AATTC | p.E746_A750>IP |
|  | 19 | c.2239_2251>C | c.2239_2251>C |
|  | 19 | c.2240_2251del12 | p.L747_T751>S |
|  | 19 | c.2238_2252>GCA | p.L747_T751>Q |
|  | 19 | c.2239_2252>CA | p.L747_T751>Q |
|  | 19 | c.2235_2246del12 | p.E746_E749delELRE |
|  | 19 | c.2235_2251>AATTC | p.E746_T751>IP |
|  | 20 | c.2310_2311insGGT | p.D770_N771insG |
|  | 20 | c.2319_2320insAACCCCCAC | p.H773_V774insNPH |
|  | 20 | c.2319_2320insCAC | p.H773_V774insH |
|  | 20 | c.2369C>T | p.T790M |
|  | 20 | c.2303G>T | p.S768I |
|  | 20 | c.2307_2308insGCCAGCGTG | p.V769_D770insASV |
|  | 20 | c.2309_2310AC>CCAGCGTGGAT | p.V769_D770insASV |
|  | 20 | c.2311_2312insGCGTGGACA | p.D770_N771insSVD |
|  | 21 | c.2573T>G | p.L858R |
|  | 21 | c.2573_2574TG>GT | p.L858R |
|  | 21 | c.2582T>A | p.L861Q |
| *KRAS* | 2 | 35G>C | G12A |
|  | 2 | 35G>A | G12D |
|  | 2 | 34G>C | G12R |
|  | 2 | 34G>T | G12C |
|  | 2 | 34G>A | G12S |
|  | 2 | 35G>T | G12V |
|  | 2 | 38G>A | G13D |
| *BRAF* | 15 | c.1799T>A | p.V600E |

**Table S5** Gene list of the 571-gene panel used in DNA NGS.

| **Mutation** | | | | | | | | | |
| --- | --- | --- | --- | --- | --- | --- | --- | --- | --- |
| *ABCB1* | *ABL1* | *ABL2* | *ABRAXAS1* | *ACVR1B* | *AGO1* | *AKT1* | *AKT2* | *AKT3* | *ALK* |
| *ALOX12B* | *AMER1* | *AP3B1* | *APC* | *APC2* | *APEX1* | *AR* | *ARAF* | *ARFRP1* | *ARID1A* |
| *ARID1B* | *ARID2* | *ARID5B* | *ASXL1* | *ATM* | *ATR* | *ATRX* | *AURKA* | *AURKB* | *AUTS2* |
| *AXIN1* | *AXIN2* | *AXL* | *B2M* | *BAP1* | *BARD1* | *BCL2* | *BCL2L1* | *BCL2L11* | *BCL2L2* |
| *BCL6* | *BCOR* | *BCORL1* | *BCR* | *BIRC3* | *BLK* | *BLM* | *BMP2* | *BMP4* | *BMPR1A* |
| *BRAF* | *BRCA1* | *BRCA2* | *BRD3* | *BRD4* | *BRIP1* | *BTG1* | *BTK* | *C8orf34* | *CALR* |
| *CARD11* | *CASP8* | *CBFB* | *CBL* | *CBLB* | *CCN6* | *CCND1* | *CCND2* | *CCND3* | *CCNE1* |
| *CD274* | *CD34* | *CD44* | *CD74* | *CD79A* | *CD79B* | *CD80* | *CD86* | *CDA* | *CDC73* |
| *CDH1* | *CDK12* | *CDK4* | *CDK6* | *CDK8* | *CDKN1A* | *CDKN1B* | *CDKN2A* | *CDKN2B* | *CDKN2C* |
| *CEBPA* | *CHD2* | *CHD4* | *CHEK1* | *CHEK2* | *CIC* | *CORO2A* | *CREBBP* | *CRKL* | *CRLF2* |
| *CSF1* | *CSF1R* | *CSF3R* | *CTCF* | *CTLA4* | *CTNNA1* | *CTNNB1* | *CUL3* | *CXCL8* | *CYLD* |
| *CYP19A1* | *CYP2C8* | *CYP2D6* | *DAXX* | *DCUN1D1* | *DDR1* | *DDR2* | *DICER1* | *DIS3* | *DKK3* |
| *DNMT1* | *DNMT3A* | *DOT1L* | *DPYD* | *DYNC2H1* | *EED* | *EGFR* | *EIF1AX* | *EIF4A2* | *EMSY* |
| *ENG* | *EP300* | *EPAS1* | *EPCAM* | *EPHA3* | *EPHA5* | *EPHA6* | *EPHA7* | *EPHB1* | *ERBB2* |
| *ERBB3* | *ERBB4* | *ERCC1* | *ERCC2* | *ERCC3* | *ERG* | *ERRFI1* | *ESR1* | *ETS2* | *ETV1* |
| *ETV4* | *ETV5* | *ETV6* | *EWSR1* | *EZH2* | *F2R* | *FANCA* | *FANCC* | *FANCD2* | *FANCE* |
| *FANCF* | *FANCG* | *FANCI* | *FANCL* | *FANCM* | *FAS* | *FAT1* | *FBXW7* | *FCGR2B* | *FGF10* |
| *FGF14* | *FGF19* | *FGF23* | *FGF3* | *FGF4* | *FGF6* | *FGF7* | *FGFR1* | *FGFR2* | *FGFR3* |
| *FGFR4* | *FGR* | *FH* | *FLCN* | *FLT1* | *FLT3* | *FLT4* | *FOXA1* | *FOXL2* | *FOXO1* |
| *FOXP1* | *FRS2* | *FUBP1* | *FUS* | *FYN* | *GABRA6* | *GATA1* | *GATA2* | *GATA3* | *GATA4* |
| *GATA6* | *GEN1* | *GLI1* | *GNA11* | *GNA13* | *GNAQ* | *GNAS* | *GREM1* | *GRIN2A* | *GRM3* |
| *GSK3B* | *GSTP1* | *H1-2* | *H2BC5* | *H3-3A* | *H3-5* | *H3C2* | *HAMP* | *HAVCR2* | *HCK* |
| *HDAC2* | *HEY1* | *HGF* | *HIF1A* | *HLA-A* | *HLA-B* | *HLA-C* | *HNF1A* | *HOXB13* | *HRAS* |
| *HSD3B1* | *HSP90AA1* | *HSPB1* | *ICOS* | *ICOSLG* | *IDH1* | *IDH2* | *IFNGR1* | *IFNGR2* | *IGF1* |
| *IGF1R* | *IGF2* | *IKBKE* | *IKZF1* | *IL13* | *IL1A* | *IL4* | *IL6* | *IL7R* | *INHBA* |
| *INPP4A* | *INPP4B* | *INSR* | *IP6K1* | *IRF1* | *IRF2* | *IRF4* | *IRS2* | *ITGB2* | *ITGB6* |
| *JAK1* | *JAK2* | *JAK3* | *JUN* | *KDM5A* | *KDM5C* | *KDM6A* | *KDR* | *KEAP1* | *KEL* |
| *KIT* | *KLF4* | *KLHL6* | *KMT2A* | *KMT2B* | *KMT2C* | *KMT2D* | *KRAS* | *LAG3* | *LATS1* |
| *LATS2* | *LCK* | *LGALS3* | *LIG4* | *LIN28B* | *LMO1* | *LRP1B* | *LYN* | *LZTR1* | *MAGI2* |
| *MAP2K1* | *MAP2K2* | *MAP2K4* | *MAP3K1* | *MAP3K13* | *MAP3K14* | *MAPK1* | *MAPK3* | *MAPK4* | *MAX* |
| *MCL1* | *MDC1* | *MDM2* | *MDM4* | *MED12* | *MEF2B* | *MEN1* | *MET* | *MGA* | *MGME1* |
| *MGMT* | *MIF* | *MITF* | *MKI67* | *MLH1* | *MLH3* | *MMP1* | *MMP7* | *MPL* | *MPO* |
| *MRE11* | *MSH2* | *MSH3* | *MSH6* | *MST1R* | *MT2A* | *MTHFR* | *MTOR* | *MTRR* | *MUC16* |
| *MUC5B* | *MUTYH* | *MYB* | *MYC* | *MYCL* | *MYCN* | *MYD88* | *MYOD1* | *NAA11* | *NAB2* |
| *NBN* | *NCOA2* | *NCOA3* | *NCOR1* | *NEIL1* | *NF1* | *NF2* | *NFE2L2* | *NFKB1* | *NFKBIA* |
| *NKX2-1* | *NOS2* | *NOS3* | *NOTCH1* | *NOTCH2* | *NOTCH3* | *NOTCH4* | *NPM1* | *NQO1* | *NR1I2* |
| *NR4A3* | *NRAS* | *NRG1* | *NSD1* | *NTRK1* | *NTRK2* | *NTRK3* | *NUP93* | *NUTM1* | *OXSR1* |
| *PAK1* | *PAK3* | *PAK5* | *PALB2* | *PAPPA2* | *PARP1* | *PAX3* | *PAX5* | *PAX7* | *PAX8* |
| *PBRM1* | *PDCD1* | *PDCD1LG2* | *PDGFB* | *PDGFRA* | *PDGFRB* | *PDK1* | *PDPK1* | *PEG3* | *PGR* |
| *PHF6* | *PIK3C2B* | *PIK3C2G* | *PIK3C3* | *PIK3CA* | *PIK3CB* | *PIK3CD* | *PIK3CG* | *PIK3R1* | *PIK3R2* |
| *PIM1* | *PLCG2* | *PLK2* | *PMS1* | *PMS2* | *PNRC1* | *POLD1* | *POLE* | *POLE4* | *PPARG* |
| *PPP2R1A* | *PPP2R2A* | *PRDM1* | *PRDX1* | *PRDX6* | *PREX2* | *PRKAA1* | *PRKACA* | *PRKAR1A* | *PRKCI* |
| *PRKDC* | *PRKN* | *PRSS8* | *PSMD4* | *PTCH1* | *PTEN* | *PTGS2* | *PTPN11* | *PTPRD* | *PTTG1* |
| *PXDNL* | *QKI* | *RAC1* | *RAD21* | *RAD50* | *RAD51* | *RAD51B* | *RAD51C* | *RAD51D* | *RAD52* |
| *RAD54L* | *RAF1* | *RANBP2* | *RARA* | *RASA1* | *RASAL1* | *RB1* | *RBM10* | *RECQL* | *RECQL4* |
| *REL* | *RET* | *REV3L* | *RHEB* | *RHOA* | *RICTOR* | *RIPK4* | *RIT1* | *RNASEL* | *RNF43* |
| *ROBO2* | *ROS1* | *RPPH1* | *RPS6KB1* | *RPS6KB2* | *RPTOR* | *RSF1* | *RUNX1* | *RUNX1T1* | *SCN8A* |
| *SDHA* | *SDHAF2* | *SDHB* | *SDHC* | *SDHD* | *SEMA3C* | *SERPINB3* | *SERPINB4* | *SERPINE1* | *SETBP1* |
| *SETD2* | *SF3B1* | *SIK1* | *SKP2* | *SLC28A3* | *SLC47A1* | *SLCO1B1* | *SLIT2* | *SLX4* | *SMAD2* |
| *SMAD3* | *SMAD4* | *SMARCA4* | *SMARCB1* | *SMARCD1* | *SMO* | *SNCAIP* | *SOCS1* | *SOD2* | *SOX10* |
| *SOX17* | *SOX2* | *SOX9* | *SPEN* | *SPOP* | *SPTA1* | *SRC* | *SRSF2* | *SS18* | *STAG2* |
| *STAT3* | *STAT4* | *STAT6* | *STK11* | *SUFU* | *SUZ12* | *SYK* | *TAF1* | *TAOK1* | *TBX3* |
| *TCF7L1* | *TCF7L2* | *TENT5C* | *TERT* | *TET1* | *TET2* | *TFE3* | *TGFB1* | *TGFBR2* | *TIGIT* |
| *TMEM127* | *TMPRSS2* | *TNF* | *TNFAIP3* | *TNFRSF14* | *TNFRSF18* | *TNFRSF9* | *TOP1* | *TOP2A* | *TP53* |
| *TPMT* | *TRAF3* | *TRAF7* | *TRRAP* | *TSC1* | *TSC2* | *TSHR* | *TXNRD2* | *TYMS* | *U2AF1* |
| *UGT1A1* | *UMPS* | *VEGFA* | *VHL* | *WRN* | *WT1* | *XPC* | *XPO1* | *XRCC1* | *XRCC2* |
| *XRCC3* | *XRCC4* | *XRCC5* | *YES1* | *YWHAE* | *ZBTB2* | *ZFHX4* | *ZNF217* | *ZNF703* | *ZNRF3* |
| *ZRSR2* |  |  |  |  |  |  |  |  |  |

| **Fusion** | | | | | | | | | |
| --- | --- | --- | --- | --- | --- | --- | --- | --- | --- |
| *ALK* | *AR* | *BRAF* | *CD74* | *EGFR* | *ERBB2* | *ERBB4* | *ESR1* | *ETV1* | *ETV4* |
| *ETV5* | *ETV6* | *EWSR1* | *FGFR1* | *FGFR2* | *FGFR3* | *FGFR4* | *FUS* | *HEY1* | *KIT* |
| *MET* | *MYB* | *NAB2* | *NCOA2* | *NOTCH2* | *NR4A3* | *NRG1* | *NTRK1* | *NTRK2* | *NTRK3* |
| *NUTM1* | *PAX3* | *PAX7* | *PAX8* | *PDGFB* | *PDGFRA* | *PDGFRB* | *RAF1* | *RET* | *ROS1* |
| *SS18* | *STAT6* | *TERT* | *TFE3* | *TMPRSS2* | *YWHAE* |  |  |  |  |

| **Copy number variant** | | | | | | | | | |
| --- | --- | --- | --- | --- | --- | --- | --- | --- | --- |
| *AKT2* | *AKT3* | *AURKA* | *CCND1* | *CCNE1* | *CD274* | *CDK4* | *CDK6* | *EGFR* | *ERBB2* |
| *FGF19* | *FGF3* | *FGFR1* | *FGFR2* | *FGFR3* | *HGF* | *IGF1R* | *MAPK1* | *MDM2* | *MDM4* |
| *MET* | *MYC* | *NTRK3* | *PDGFRA* | *PGR* | *PIK3CA* | *RET* | *RICTOR* | *SMO* | *TOP2A* |

**Table S6** Gene list of the 2660-gene panel used in RNA NGS.

| **Fusion** | | | | | | | | | |
| --- | --- | --- | --- | --- | --- | --- | --- | --- | --- |
| *ALK* | *AR* | *BRAF* | *CD74* | *CLDN18* | *EGFR* | *ERBB2* | *ERBB4* | *ESR1* | *ETV1* |
| *ETV4* | *ETV5* | *ETV6* | *EWSR1* | *FGFR1* | *FGFR2* | *FGFR3* | *FGFR4* | *HEY1* | *KIT* |
| *MET* | *MYB* | *NAB2* | *NCOA2* | *NOTCH2* | *NR4A3* | *NRG1* | *NRG2* | *NRG3* | *NTRK1* |
| *NTRK2* | *NTRK3* | *NUTM1* | *PAX8* | *PDGFB* | *PDGFRA* | *PDGFRB* | *RAF1* | *RET* | *ROS1* |
| *SS18* | *STAT6* | *TFE3* | *TMPRSS2* | *YWHAE* |  |  |  |  |  |

| **Gene expression** | | | | | | | | | |
| --- | --- | --- | --- | --- | --- | --- | --- | --- | --- |
| *A2M* | *ABCB1* | *ABCF1* | *ABL1* | *ABR* | *ABTB2* | *ACAD9* | *ACADM* | *ACAN* | *ACOT12* |
| *ACSF3* | *ACTA2* | *ACTB* | *ACTG1* | *ACTG2* | *ACTL6A* | *ACTL6B* | *ACTR3B* | *ACVR1B* | *ACVR1C* |
| *ACVR2A* | *ACY1* | *ADA* | *ADAM12* | *ADAMTS16* | *ADGRE1* | *ADGRE2* | *ADGRE5* | *ADH1A* | *ADH1B* |
| *ADH1C* | *ADH4* | *ADH6* | *ADM* | *ADORA2A* | *AFAP1* | *AFDN* | *AFF3* | *AGAP3* | *AGBL4* |
| *AGGF1* | *AGK* | *AGR2* | *AGTRAP* | *AHCYL1* | *AHR* | *AICDA* | *AIF1* | *AIRE* | *AKAP1* |
| *AKAP13* | *AKAP9* | *AKR1C3* | *AKR1C4* | *AKT1* | *AKT2* | *AKT3* | *ALAD* | *ALAS1* | *ALCAM* |
| *ALDOA* | *ALDOC* | *ALK* | *ALKBH2* | *ALKBH3* | *ALOX15B* | *AMBP* | *AMBRA1* | *AMER1* | *AMH* |
| *AMMECR1L* | *AMOT* | *AMOTL2* | *ANGPT1* | *ANGPT2* | *ANGPTL4* | *ANKLE2* | *ANKRD28* | *ANKRD46* | *ANLN* |
| *ANO3* | *ANP32B* | *ANXA1* | *AP1M1* | *AP3B1* | *APAF1* | *APBB1* | *APC* | *APC2* | *APH1B* |
| *API5* | *APIP* | *APLNR* | *APOA1* | *APOA2* | *APOA4* | *APOB* | *APOBEC3B* | *APOC2* | *APOC3* |
| *APOD* | *APOE* | *APOL6* | *APOLD1* | *APOM* | *APP* | *APPL1* | *AQP9* | *AR* | *ARAF* |
| *AREG* | *ARF1* | *ARG1* | *ARG2* | *ARHGEF2* | *ARHGEF6* | *ARID1A* | *ARID1B* | *ARID2* | *ARID5A* |
| *ARMC10* | *ARMH3* | *ARNT* | *ARNT2* | *ARNTL* | *ASAP2* | *ASCL1* | *ASL* | *ASNS* | *ASPA* |
| *ASPG* | *ASPN* | *ASPSCR1* | *ASXL1* | *ATF1* | *ATF2* | *ATF3* | *ATF4* | *ATF7IP* | *ATG10* |
| *ATG12* | *ATG16L1* | *ATG5* | *ATG7* | *ATIC* | *ATM* | *ATOX1* | *ATP11C* | *ATP1B1* | *ATP2A2* |
| *ATP5F1D* | *ATP5F1E* | *ATP5ME* | *ATP6V1D* | *ATR* | *ATRX* | *AURKA* | *AURKB* | *AXIN1* | *AXIN2* |
| *AXL* | *AZGP1* | *B2M* | *B3GAT1* | *B4GALT6* | *BACH2* | *BAD* | *BAG1* | *BAG4* | *BAIAP2L1* |
| *BAIAP3* | *BAK1* | *BAMBI* | *BAP1* | *BATF* | *BATF3* | *BAX* | *BBC3* | *BBS1* | *BCAN* |
| *BCAT1* | *BCL10* | *BCL11B* | *BCL2* | *BCL2A1* | *BCL2L1* | *BCL2L11* | *BCL2L14* | *BCL3* | *BCL6* |
| *BCL6B* | *BCOR* | *BCR* | *BDNF* | *BGN* | *BICC1* | *BID* | *BIRC2* | *BIRC3* | *BIRC5* |
| *BIRC7* | *BLK* | *BLM* | *BLNK* | *BLVRA* | *BMI1* | *BMP2* | *BMP4* | *BMP5* | *BMP6* |
| *BMP7* | *BMP8A* | *BMPR1B* | *BNIP3* | *BNIP3L* | *BRAF* | *BRCA1* | *BRCA2* | *BRD2* | *BRD3* |
| *BRD4* | *BRD7* | *BRIP1* | *BRIX1* | *BST1* | *BST2* | *BTBD1* | *BTF3L4* | *BTK* | *BTLA* |
| *BUB1* | *BUB1B-PAK6* | *BYSL* | *C1QA* | *C1QB* | *C1QBP* | *C1R* | *C1S* | *C2* | *C2CD5* |
| *C3* | *C3AR1* | *C4B* | *C4BPA* | *C5* | *C5AR1* | *C6* | *C7* | *C8A* | *C8B* |
| *C8G* | *C8orf34* | *C9* | *CA12* | *CA2* | *CA4* | *CACNA1C* | *CACNA1D* | *CACNA1E* | *CACNA1G* |
| *CACNA1H* | *CACNA2D1* | *CACNA2D2* | *CACNA2D3* | *CACNA2D4* | *CACNB2* | *CACNB3* | *CACNB4* | *CACNG1* | *CACNG4* |
| *CACNG6* | *CADPS* | *CALM1* | *CALM2* | *CALM3* | *CALML3* | *CALML4* | *CALML5* | *CALML6* | *CAMK1* |
| *CAMK1D* | *CAMK1G* | *CAMK2A* | *CAMK2B* | *CAMK2D* | *CAMK2G* | *CAMK4* | *CAMP* | *CAPN2* | *CAPN6* |
| *CAPZA2* | *CARD11* | *CARD9* | *CASP1* | *CASP10* | *CASP12* | *CASP3* | *CASP7* | *CASP8* | *CASP9* |
| *CAV1* | *CBL* | *CBLB* | *CBLC* | *CBR4* | *CBX5* | *CC2D1B* | *CCAR2* | *CCDC186* | *CCDC198* |
| *CCDC6* | *CCDC91* | *CCL1* | *CCL11* | *CCL13* | *CCL14* | *CCL15* | *CCL16* | *CCL17* | *CCL18* |
| *CCL19* | *CCL2* | *CCL20* | *CCL21* | *CCL22* | *CCL23* | *CCL24* | *CCL25* | *CCL26* | *CCL27* |
| *CCL28* | *CCL3* | *CCL3L1* | *CCL4* | *CCL5* | *CCL7* | *CCL8* | *CCNA1* | *CCNA2* | *CCNB1* |
| *CCNB2* | *CCNB3* | *CCND1* | *CCND2* | *CCND3* | *CCNE1* | *CCNE2* | *CCNO* | *CCR1* | *CCR2* |
| *CCR3* | *CCR4* | *CCR5* | *CCR6* | *CCR7* | *CCR9* | *CCRL2* | *CD101* | *CD14* | *CD160* |
| *CD163* | *CD164* | *CD180* | *CD19* | *CD1A* | *CD1B* | *CD1C* | *CD1D* | *CD1E* | *CD2* |
| *CD200* | *CD207* | *CD209* | *CD22* | *CD226* | *CD24* | *CD244* | *CD247* | *CD27* | *CD274* |
| *CD276* | *CD28* | *CD300A* | *CD33* | *CD34* | *CD36* | *CD37* | *CD38* | *CD3D* | *CD3E* |
| *CD3G* | *CD4* | *CD40* | *CD40LG* | *CD44* | *CD46* | *CD47* | *CD48* | *CD5* | *CD52* |
| *CD53* | *CD55* | *CD58* | *CD59* | *CD6* | *CD63* | *CD68* | *CD69* | *CD7* | *CD70* |
| *CD74* | *CD79A* | *CD79B* | *CD80* | *CD81* | *CD83* | *CD84* | *CD86* | *CD8A* | *CD8B* |
| *CD9* | *CD96* | *CD99* | *CDC14A* | *CDC14B* | *CDC20* | *CDC25A* | *CDC25B* | *CDC25C* | *CDC27* |
| *CDC42* | *CDC42EP1* | *CDC6* | *CDC7* | *CDCA3* | *CDH1* | *CDH11* | *CDH16* | *CDH17* | *CDH2* |
| *CDH3* | *CDH5* | *CDK1* | *CDK12* | *CDK2* | *CDK4* | *CDK6* | *CDKN1A* | *CDKN1B* | *CDKN1C* |
| *CDKN2A* | *CDKN2B* | *CDKN2C* | *CDKN2D* | *CDKN3* | *CDX2* | *CEACAM1* | *CEACAM3* | *CEACAM5* | *CEACAM6* |
| *CEACAM8* | *CEBPA* | *CEBPB* | *CEBPE* | *CELSR2* | *CENPF* | *CEP43* | *CEP55* | *CEP72* | *CEP85L* |
| *CEP89* | *CES3* | *CFB* | *CFD* | *CFI* | *CFL1* | *CFP* | *CGAS* | *CHAD* | *CHEK1* |
| *CHEK2* | *CHGA* | *CHI3L1* | *CHIT1* | *CHRM3* | *CHSY1* | *CHTOP* | *CHUK* | *CIC* | *CIDEA* |
| *CIITA* | *CIT* | *CKLF* | *CKS1B* | *CKS2* | *CLCF1* | *CLCN6* | *CLDN18* | *CLEC10A* | *CLEC14A* |
| *CLEC4A* | *CLEC4C* | *CLEC4E* | *CLEC5A* | *CLEC6A* | *CLEC7A* | *CLECL1* | *CLIP1* | *CLTC* | *CLU* |
| *CMA1* | *CMKLR1* | *CMTM4* | *CMTM6* | *CNIH4* | *CNOT10* | *CNOT2* | *CNOT4* | *CNTFR* | *CNTRL* |
| *COG7* | *COL11A1* | *COL11A2* | *COL14A1* | *COL16A1* | *COL17A1* | *COL1A1* | *COL1A2* | *COL24A1* | *COL27A1* |
| *COL2A1* | *COL3A1* | *COL4A1* | *COL4A2* | *COL4A3* | *COL4A4* | *COL4A5* | *COL4A6* | *COL5A1* | *COL5A2* |
| *COL6A3* | *COL6A6* | *COLEC12* | *COMP* | *CORO1A* | *COX11* | *COX4I1* | *COX5B* | *COX6A1* | *COX6B1* |
| *CPA3* | *CPEB2* | *CPSF7* | *CR1* | *CR2* | *CRABP2* | *CREB1* | *CREB3* | *CREB3L1* | *CREB3L2* |
| *CREB3L3* | *CREB3L4* | *CREB5* | *CREBBP* | *CRK* | *CRKL* | *CRLF2* | *CRP* | *CRTAM* | *CSF1* |
| *CSF1R* | *CSF2* | *CSF2RB* | *CSF3* | *CSF3R* | *CSNK1A1* | *CSNK1A1L* | *CST2* | *CT45A1* | *CTAG1B* |
| *CTAG2* | *CTAGE1* | *CTBP1* | *CTBP2* | *CTCFL* | *CTLA4* | *CTNNA1* | *CTNNA2* | *CTNNA3* | *CTNNB1* |
| *CTRC* | *CTSC* | *CTSG* | *CTSH* | *CTSL* | *CTSS* | *CTSV* | *CTSW* | *CTTN* | *CUL1* |
| *CUL2* | *CUL3* | *CUX1* | *CWH43* | *CX3CL1* | *CX3CR1* | *CXADR* | *CXCL1* | *CXCL10* | *CXCL11* |
| *CXCL12* | *CXCL13* | *CXCL14* | *CXCL16* | *CXCL2* | *CXCL3* | *CXCL5* | *CXCL6* | *CXCL8* | *CXCL9* |
| *CXCR1* | *CXCR2* | *CXCR3* | *CXCR4* | *CXCR5* | *CXCR6* | *CXXC4* | *CXXC5* | *CYB561* | *CYBB* |
| *CYCS* | *CYFIP2* | *CYLD* | *CYP17A1* | *CYP19A1* | *CYP1B1* | *CYP2D6* | *CYP4A11* | *CYP4A22* | *CYP8B1* |
| *CYSTM1* | *DAB2* | *DACH2* | *DAPK1* | *DAPK2* | *DAPK3* | *DAXX* | *DCC* | *DCSTAMP* | *DCTN1* |
| *DDB1* | *DDB2* | *DDIT3* | *DDIT4* | *DDX21* | *DDX43* | *DDX50* | *DDX58* | *DEFB1* | *DEFB134* |
| *DEPTOR* | *DGAT2* | *DGCR2* | *DGLUCY* | *DHX15* | *DHX16* | *DIAPH1* | *DIO1* | *DIO2* | *DIPK2B* |
| *DKK1* | *DKK2* | *DKK4* | *DLK1* | *DLL1* | *DLL3* | *DLL4* | *DLX2* | *DMBT1* | *DNAJC14* |
| *DNMT1* | *DNMT3A* | *DOCK9* | *DPF1* | *DPF3* | *DPP4* | *DSC3* | *DSP* | *DST* | *DTX1* |
| *DTX3* | *DTX3L* | *DTX4* | *DUOX1* | *DUOX2* | *DUSP1* | *DUSP10* | *DUSP2* | *DUSP4* | *DUSP5* |
| *DUSP6* | *DUSP8* | *DVL1* | *DVL2* | *DVL3* | *DYNC1I2* | *DZANK1* | *E2F1* | *E2F2* | *E2F3* |
| *E2F4* | *E2F5* | *EBI3* | *ECSIT* | *EDC3* | *EDN1* | *EEF1G* | *EFNA1* | *EFNA2* | *EFNA3* |
| *EFNA4* | *EFNA5* | *EGF* | *EGFR* | *EGLN1* | *EGLN2* | *EGLN3* | *EGR1* | *EGR2* | *EGR3* |
| *EHHADH* | *EIF1* | *EIF2AK2* | *EIF2AK3* | *EIF2B4* | *EIF3L* | *EIF4A2* | *EIF4EBP1* | *EIF5AL1* | *ELANE* |
| *ELAVL3* | *ELAVL4* | *ELK1* | *ELMO1* | *ELOB* | *ELOC* | *ELOVL6* | *EML4* | *EMX2* | *ENDOG* |
| *ENG* | *ENO1* | *ENTPD1* | *EOMES* | *EP300* | *EPAS1* | *EPCAM* | *EPHA2* | *EPM2AIP1* | *EPO* |
| *EPOR* | *EPS15* | *EPS8L3* | *ERBB2* | *ERBB4* | *ERC1* | *ERCC1* | *ERCC2* | *ERCC3* | *ERCC4* |
| *ERCC5* | *ERCC6* | *EREG* | *ERG* | *ERLIN2* | *ERN2* | *ERO1A* | *ERP44* | *ESR1* | *ESR2* |
| *ETHE1* | *ETS1* | *ETS2* | *ETV1* | *ETV4* | *ETV5* | *ETV6* | *ETV7* | *EVA1A* | *EWSR1* |
| *EXO1* | *EYA1* | *EZH2* | *EZR* | *F11* | *F11R* | *F12* | *F13A1* | *F2RL1* | *FAAP24* |
| *FABP1* | *FABP4* | *FADD* | *FAM114A2* | *FAM124B* | *FAM131B* | *FAM13C* | *FAM167A* | *FAM30A* | *FANCA* |
| *FANCB* | *FANCC* | *FANCD2* | *FANCE* | *FANCF* | *FANCG* | *FANCL* | *FAP* | *FAS* | *FASLG* |
| *FAU* | *FBP1* | *FBXO28* | *FBXW7* | *FCAR* | *FCER1A* | *FCER1G* | *FCER2* | *FCF1* | *FCGR1A* |
| *FCGR2A* | *FCGR2B* | *FCGR3A* | *FCGR3B* | *FCGRT* | *FCHO1* | *FCHSD1* | *FCN1* | *FCRL2* | *FCRLA* |
| *FEN1* | *FEZ1* | *FGF1* | *FGF10* | *FGF11* | *FGF12* | *FGF13* | *FGF14* | *FGF16* | *FGF17* |
| *FGF18* | *FGF19* | *FGF2* | *FGF20* | *FGF21* | *FGF22* | *FGF23* | *FGF3* | *FGF4* | *FGF5* |
| *FGF6* | *FGF7* | *FGF8* | *FGF9* | *FGFR1* | *FGFR1OP2* | *FGFR2* | *FGFR3* | *FGFR4* | *FH* |
| *FHIT* | *FILIP1* | *FIP1L1* | *FKBP15* | *FLCN* | *FLG* | *FLI1* | *FLNA* | *FLNB* | *FLNC* |
| *FLT1* | *FLT3* | *FLT3LG* | *FLT4* | *FN1* | *FOLH1* | *FOS* | *FOSL1* | *FOXA1* | *FOXA2* |
| *FOXC1* | *FOXE1* | *FOXG1* | *FOXJ1* | *FOXL2* | *FOXM1* | *FOXO1* | *FOXO3* | *FOXO4* | *FOXP3* |
| *FPR1* | *FPR2* | *FPR3* | *FRAT1* | *FRAT2* | *FST* | *FSTL3* | *FUBP1* | *FUT4* | *FUT5* |
| *FUT7* | *FUT8* | *FYB1* | *FYN* | *FZD1* | *FZD10* | *FZD2* | *FZD3* | *FZD4* | *FZD5* |
| *FZD6* | *FZD7* | *FZD8* | *FZD9* | *G6PD* | *GAB1* | *GAB2* | *GABPA* | *GABRB2* | *GADD45A* |
| *GADD45B* | *GADD45G* | *GADD45GIP1* | *GADL1* | *GAGE1* | *GAGE10* | *GAGE12F* | *GAGE12I* | *GAGE12J* | *GAGE13* |
| *GAGE2A* | *GAGE2C* | *GAGE2E* | *GAPDH* | *GAS1* | *GATA1* | *GATA2* | *GATA3* | *GBP1* | *GBP2* |
| *GBP4* | *GCG* | *GCGR* | *GDF15* | *GDF6* | *GEMIN4* | *GFAP* | *GHITM* | *GHR* | *GIMAP4* |
| *GIMAP6* | *GIT2* | *GJA1* | *GJB6* | *GKAP1* | *GLI1* | *GLI2* | *GLI3* | *GLIS3* | *GLOD4* |
| *GLS* | *GLUD1* | *GLUL* | *GMIP* | *GNA11* | *GNA14* | *GNAQ* | *GNAS* | *GNG12* | *GNG4* |
| *GNG7* | *GNGT1* | *GNL3* | *GNLY* | *GOLGA5* | *GOPC* | *GOT1* | *GOT2* | *GPATCH3* | *GPC4* |
| *GPI* | *GPM6B* | *GPR160* | *GPR18* | *GPR3* | *GPS1* | *GPSM3* | *GPT* | *GPX1* | *GPX3* |
| *GPX4* | *GRAP2* | *GRB10* | *GRB2* | *GRB7* | *GREM1* | *GRIA3* | *GRIN1* | *GRIN2A* | *GRIN2B* |
| *GRIPAP1* | *GSK3B* | *GSN* | *GSTA1* | *GSTA2* | *GSTA3* | *GSTA4* | *GSTA5* | *GSTM1* | *GSTM2* |
| *GSTM3* | *GSTM4* | *GSTM5* | *GSTO1* | *GSTO2* | *GSTP1* | *GSTT1* | *GSTT2* | *GSTT2B* | *GTF2H3* |
| *GTF2I* | *GTF2IRD1* | *GTF3C1* | *GTPBP4* | *GUSB* | *GYG1* | *GZMA* | *GZMB* | *GZMH* | *GZMK* |
| *GZMM* | *H2AX* | *H3-3A* | *H3-5* | *H3C10* | *H3C2* | *H3C8* | *HACD2* | *HAMP* | *HAVCR2* |
| *HBB* | *HBEGF* | *HCK* | *HDAC1* | *HDAC10* | *HDAC11* | *HDAC2* | *HDAC3* | *HDAC4* | *HDAC5* |
| *HDAC6* | *HDC* | *HELLS* | *HERC6* | *HES1* | *HES5* | *HEY1* | *HEY2* | *HEYL* | *HFM1* |
| *HGD* | *HGF* | *HHEX* | *HHIP* | *HIF1A* | *HIP1* | *HK1* | *HK2* | *HLA-A* | *HLA-B* |
| *HLA-C* | *HLA-DMA* | *HLA-DMB* | *HLA-DOA* | *HLA-DOB* | *HLA-DPA1* | *HLA-DPB1* | *HLA-DQA1* | *HLA-DQA2* | *HLA-DQB1* |
| *HLA-DQB2* | *HLA-DRA* | *HLA-DRB1* | *HLA-DRB3* | *HLA-DRB4* | *HLA-DRB5* | *HLA-E* | *HLA-F* | *HLA-F-AS1* | *HLA-G* |
| *HLF* | *HMBS* | *HMGA1* | *HMGA2* | *HMGB1* | *HMGN5* | *HMOX1* | *HNF1A* | *HNRNPA2B1* | *HNRNPL* |
| *HOXA10* | *HOXA11* | *HOXA9* | *HOXC10* | *HOXD11* | *HPGD* | *HPRT1* | *HRAS* | *HSD11B1* | *HSD17B8* |
| *HSDL2* | *HSF2BP* | *HSP90AA1* | *HSP90AB1* | *HSP90B1* | *HSPA1A* | *HSPA2* | *HSPA6* | *HSPB1* | *HTR3A* |
| *HYDIN* | *IBSP* | *ICAM1* | *ICAM2* | *ICAM3* | *ICAM4* | *ICAM5* | *ICOS* | *ICOSLG* | *ID1* |
| *ID2* | *ID3* | *ID4* | *IDH1* | *IDH2* | *IDO1* | *IDO2* | *IER3* | *IFI16* | *IFI27* |
| *IFI35* | *IFI44L* | *IFI6* | *IFIH1* | *IFIT1* | *IFIT2* | *IFIT3* | *IFITM1* | *IFITM2* | *IFNA1* |
| *IFNA17* | *IFNA2* | *IFNA7* | *IFNA8* | *IFNAR1* | *IFNAR2* | *IFNB1* | *IFNG* | *IFNGR1* | *IFNGR2* |
| *IFNL1* | *IFNL2* | *IGF1* | *IGF1R* | *IGF2* | *IGF2R* | *IGFBP2* | *IGFBP3* | *IGFBP7* | *IGLL1* |
| *IGSF6* | *IHH* | *IKBKB* | *IKBKE* | *IKBKG* | *IKZF1* | *IKZF2* | *IKZF3* | *IKZF4* | *IL10* |
| *IL10RA* | *IL11* | *IL11RA* | *IL12A* | *IL12B* | *IL12RB1* | *IL12RB2* | *IL13* | *IL13RA1* | *IL13RA2* |
| *IL15* | *IL15RA* | *IL16* | *IL17A* | *IL17B* | *IL17F* | *IL17RA* | *IL17RB* | *IL18* | *IL18R1* |
| *IL18RAP* | *IL19* | *IL1A* | *IL1B* | *IL1R1* | *IL1R2* | *IL1RAP* | *IL1RAPL2* | *IL1RL1* | *IL1RL2* |
| *IL1RN* | *IL2* | *IL20RA* | *IL20RB* | *IL21* | *IL21R* | *IL22* | *IL22RA1* | *IL22RA2* | *IL23A* |
| *IL23R* | *IL24* | *IL25* | *IL26* | *IL27* | *IL2RA* | *IL2RB* | *IL2RG* | *IL3* | *IL32* |
| *IL33* | *IL34* | *IL3RA* | *IL4* | *IL4R* | *IL5* | *IL5RA* | *IL6* | *IL6R* | *IL6ST* |
| *IL7* | *IL7R* | *IL9* | *ILF3* | *ILK* | *ING4* | *INHBA* | *INHBB* | *INPP5D* | *INS* |
| *INSL4* | *INSRR* | *IRAK1* | *IRAK2* | *IRAK3* | *IRAK4* | *IRF1* | *IRF2* | *IRF2BP2* | *IRF3* |
| *IRF4* | *IRF5* | *IRF7* | *IRF8* | *IRF9* | *IRGM* | *IRS1* | *ISG15* | *ISG20* | *ISL1* |
| *ITCH* | *ITGA1* | *ITGA2* | *ITGA2B* | *ITGA3* | *ITGA4* | *ITGA5* | *ITGA6* | *ITGA7* | *ITGA8* |
| *ITGA9* | *ITGAE* | *ITGAL* | *ITGAM* | *ITGAV* | *ITGAX* | *ITGB1* | *ITGB2* | *ITGB3* | *ITGB4* |
| *ITGB6* | *ITGB7* | *ITGB8* | *ITK* | *ITPK1* | *JADE1* | *JAG1* | *JAG2* | *JAK1* | *JAK2* |
| *JAK3* | *JAKMIP1* | *JAM3* | *JAML* | *JCHAIN* | *JUN* | *JUNB* | *JUP* | *KAT2B* | *KATNAL2* |
| *KBTBD8* | *KCNAB1* | *KCNIP3* | *KCNJ11* | *KCNN4* | *KCTD8* | *KDELR2* | *KDM5C* | *KDM6A* | *KDM7A* |
| *KDR* | *KEAP1* | *KIAA1217* | *KIAA1549* | *KIAA1598* | *KIF12* | *KIF2C* | *KIF5B* | *KIF7* | *KIR2DL1* |
| *KIR2DL2* | *KIR2DL3* | *KIR2DS4* | *KIR3DL1* | *KIR3DL2* | *KIR3DL3* | *KIR3DS1* | *KIT* | *KITLG* | *KLC1* |
| *KLF2* | *KLF4* | *KLHL7* | *KLK2* | *KLK3* | *KLRB1* | *KLRC1* | *KLRC2* | *KLRD1* | *KLRF1* |
| *KLRG1* | *KLRK1* | *KMT2C* | *KMT2D* | *KRAS* | *KREMEN1* | *KRT1* | *KRT10* | *KRT13* | *KRT14* |
| *KRT15* | *KRT17* | *KRT18* | *KRT19* | *KRT20* | *KRT5* | *KRT6A* | *KRT6B* | *KRT6C* | *KRT7* |
| *KYAT1* | *L1CAM* | *LAG3* | *LAIR1* | *LAIR2* | *LAMA1* | *LAMA2* | *LAMA3* | *LAMA4* | *LAMA5* |
| *LAMB1* | *LAMB2* | *LAMB3* | *LAMB4* | *LAMC1* | *LAMC2* | *LAMC3* | *LAMP1* | *LAMP2* | *LAMP3* |
| *LAPTM5* | *LAT* | *LBP* | *LCK* | *LCN2* | *LCOR* | *LCP1* | *LDHA* | *LDHB* | *LEF1* |
| *LEFTY1* | *LEFTY2* | *LEP* | *LEPR* | *LEXM* | *LFNG* | *LGALS3* | *LGALS4* | *LGALS9* | *LGR5* |
| *LHX3* | *LIF* | *LIFR* | *LIG1* | *LIG3* | *LIG4* | *LILRA1* | *LILRA4* | *LILRA5* | *LILRB1* |
| *LILRB2* | *LILRB3* | *LILRB4* | *LIMA1* | *LLGL1* | *LMNA* | *LOH12CR1* | *LOXL2* | *LRG1* | *LRP1* |
| *LRP2* | *LRP5* | *LRP6* | *LRRC32* | *LRRC71* | *LRRN3* | *LSM12* | *LSM14A* | *LST1* | *LTA* |
| *LTB* | *LTBP1* | *LTBR* | *LTF* | *LTK* | *LUC7L2* | *LUM* | *LY6E* | *LY6K* | *LY86* |
| *LY9* | *LY96* | *LYN* | *LYZ* | *LZTFL1* | *M6PR* | *MAD1L1* | *MAD2L1* | *MAD2L2* | *MADCAM1* |
| *MAF* | *MAFF* | *MAGEA1* | *MAGEA10* | *MAGEA12* | *MAGEA3* | *MAGEA4* | *MAGEA6* | *MAGEB2* | *MAGEC1* |
| *MAGEC2* | *MAGI3* | *MALT1* | *MAML2* | *MAP2K1* | *MAP2K2* | *MAP2K3* | *MAP2K4* | *MAP2K6* | *MAP3K1* |
| *MAP3K12* | *MAP3K13* | *MAP3K14* | *MAP3K20* | *MAP3K5* | *MAP3K7* | *MAP3K8* | *MAP4K2* | *MAPK1* | *MAPK10* |
| *MAPK11* | *MAPK12* | *MAPK14* | *MAPK3* | *MAPK8* | *MAPK8IP1* | *MAPK8IP2* | *MAPK9* | *MAPKAPK2* | *MAPT* |
| *MARCKS* | *MARCO* | *MASP1* | *MASP2* | *MAVS* | *MAX* | *MBL2* | *MBNL1* | *MBNL3* | *MCAM* |
| *MCAT* | *MCL1* | *MCM2* | *MCM4* | *MCM5* | *MCM7* | *MDC1* | *MDFIC* | *MDM2* | *MDM4* |
| *ME2* | *MECOM* | *MED12* | *MEF2C* | *MEF2D* | *MEFV* | *MEIS1* | *MELK* | *MEN1* | *MERTK* |
| *MET* | *MFGE8* | *MFNG* | *MGEA5* | *MGMT* | *MGP* | *MGST1* | *MGST2* | *MGST3* | *MIA* |
| *MIB1* | *MICA* | *MICB* | *MIF* | *MITF* | *MKI67* | *MKRN1* | *MLANA* | *MLEC* | *MLF1* |
| *MLH1* | *MLLT10* | *MLLT3* | *MLPH* | *MME* | *MMP1* | *MMP11* | *MMP12* | *MMP2* | *MMP3* |
| *MMP7* | *MMP9* | *MMRN2* | *MNAT1* | *MNX1* | *MORC3* | *MPL* | *MPO* | *MPPED1* | *MPRIP* |
| *MR1* | *MRC1* | *MRE11* | *MRM2* | *MRPL19* | *MRPS5* | *MS4A1* | *MS4A2* | *MS4A4A* | *MS4A6A* |
| *MSH2* | *MSH3* | *MSH6* | *MSMB* | *MSN* | *MSR1* | *MSRB2* | *MST1R* | *MTF1* | *MTF2* |
| *MTMR14* | *MTOR* | *MTRR* | *MUC1* | *MUC2* | *MUC4* | *MUTYH* | *MX1* | *MXI1* | *MYB* |
| *MYBL2* | *MYC* | *MYCN* | *MYCT1* | *MYD88* | *MYH9* | *MYO18A* | *MYO5A* | *MYRIP* | *MZT1* |
| *NAALAD2* | *NAB2* | *NACC2* | *NANOG* | *NASP* | *NAT1* | *NAT8L* | *NBN* | *NCAM1* | *NCF1* |
| *NCF4* | *NCL* | *NCOA1* | *NCOA2* | *NCOA3* | *NCOA4* | *NCOR1* | *NCOR2* | *NCR1* | *NCR3* |
| *NDC1* | *NDC80* | *NDUFA1* | *NDUFA11* | *NDUFA12* | *NDUFA13* | *NDUFA2* | *NDUFA3* | *NDUFA4L2* | *NDUFA6* |
| *NDUFA7* | *NDUFB1* | *NDUFB10* | *NDUFB11* | *NDUFB4* | *NDUFB7* | *NDUFB8* | *NDUFS7* | *NDUFS8* | *NECTIN1* |
| *NECTIN2* | *NECTIN4* | *NEFL* | *NEIL1* | *NEIL3* | *NF1* | *NF2* | *NFAM1* | *NFASC* | *NFATC1* |
| *NFATC2* | *NFATC3* | *NFATC4* | *NFE2L2* | *NFIB* | *NFIL3* | *NFKB1* | *NFKB2* | *NFKBIA* | *NFKBIE* |
| *NFKBIZ* | *NGF* | *NGFR* | *NID2* | *NKD1* | *NKG7* | *NKX2-1* | *NKX3-1* | *NLRC5* | *NLRP3* |
| *NOD1* | *NOD2* | *NODAL* | *NOG* | *NOL4* | *NOL7* | *NOP16* | *NOS1* | *NOS1AP* | *NOS2* |
| *NOS3* | *NOTCH1* | *NOTCH2* | *NOTCH3* | *NOTCH4* | *NOX1* | *NPM1* | *NPM2* | *NPTX2* | *NPY1R* |
| *NQO1* | *NR3C1* | *NR4A1* | *NR4A3* | *NRAP* | *NRAS* | *NRBF2* | *NRDE2* | *NRG1* | *NRG2* |
| *NRG3* | *NRP1* | *NSD1* | *NSD2* | *NSD3* | *NT5E* | *NTF3* | *NTHL1* | *NTN3* | *NTRK1* |
| *NTRK2* | *NTRK3* | *NUB1* | *NUBP1* | *NUF2* | *NUMB* | *NUMBL* | *NUP107* | *NUTM1* | *NUPR1* |
| *OAS1* | *OAS2* | *OAS3* | *OASL* | *OAT* | *OAZ1* | *OCIAD1* | *OFD1* | *OLFML2B* | *OLR1* |
| *OPN3* | *ORC6* | *OSM* | *OTC* | *OTOA* | *OXR1* | *P2RY13* | *P4HA1* | *P4HA2* | *PAK1* |
| *PAK2* | *PAK3* | *PAK4* | *PAK5* | *PAK6* | *PALMD* | *PAN3* | *PANX3* | *PAPD7* | *PAPSS1* |
| *PARG* | *PARP12* | *PARP2* | *PARP4* | *PARP9* | *PASD1* | *PAWR* | *PAX3* | *PAX5* | *PAX8* |
| *PBK* | *PBRM1* | *PBX1* | *PBX3* | *PC* | *PCBP1* | *PCDH7* | *PCK1* | *PCK2* | *PCLAF* |
| *PCM1* | *PCNA* | *PCP4* | *PDCD1* | *PDCD1LG2* | *PDE5A* | *PDE7A* | *PDE9A* | *PDGFA* | *PDGFB* |
| *PDGFC* | *PDGFD* | *PDGFRA* | *PDGFRB* | *PDK1* | *PDLIM4* | *PDPK1* | *PDPN* | *PDZK1IP1* | *PEAR1* |
| *PEBP1* | *PECAM1* | *PEG3* | *PER2* | *PF4* | *PFKFB3* | *PFKM* | *PGAP3* | *PGF* | *PGK1* |
| *PGM2* | *PGPEP1* | *PGR* | *PHC3* | *PHF10* | *PHF12* | *PHF6* | *PHGDH* | *PHLDA2* | *PHLDB3* |
| *PI15* | *PIAS1* | *PIAS2* | *PIAS3* | *PIAS4* | *PIGR* | *PIK3CA* | *PIK3CB* | *PIK3CD* | *PIK3CG* |
| *PIK3R1* | *PIK3R2* | *PIK3R3* | *PIK3R4* | *PIK3R5* | *PIM1* | *PIM2* | *PIN1* | *PITX2* | *PKM* |
| *PKMYT1* | *PKP3* | *PLA1A* | *PLA2G10* | *PLA2G1B* | *PLA2G2A* | *PLA2G3* | *PLA2G4A* | *PLA2G4C* | *PLA2G4E* |
| *PLA2G4F* | *PLA2G5* | *PLA2G6* | *PLAT* | *PLAU* | *PLAUR* | *PLCB1* | *PLCB4* | *PLCD3* | *PLCE1* |
| *PLCG1* | *PLCG2* | *PLD1* | *PLD2* | *PLEKHA5* | *PLEKHG6* | *PLK1* | *PLK3* | *PLOD2* | *PMAIP1* |
| *PMCH* | *PMEL* | *PMEPA1* | *PML* | *PMS2* | *PNKP* | *PNMA1* | *PNOC* | *PNPLA5* | *POC1B* |
| *POF1B* | *POLB* | *POLD1* | *POLD2* | *POLD4* | *POLE2* | *POLK* | *POLR1B* | *POLR1C* | *CD3EAP* |
| *POLR2A* | *POLR2D* | *POLR2H* | *POLR2J* | *POLR3G* | *POSTN* | *POU2AF1* | *POU2F2* | *POU5F1* | *PPA1* |
| *PPAN* | *PPARD* | *PPARG* | *PPARGC1A* | *PPARGC1B* | *PPAT* | *PPBP* | *PPFIBP1* | *PPHLN1* | *PPIA* |
| *PPL* | *PPP1R1B* | *PPP1R21* | *PPP2CB* | *PPP2R1A* | *PPP2R2B* | *PPP2R2C* | *PPP2R3A* | *PPP3CA* | *PPP3CB* |
| *PPP3CC* | *PPP3R1* | *PPP3R2* | *PPP4R3B* | *PRAME* | *PRC1* | *PRCC* | *PRDM1* | *PRDM6* | *PRDX1* |
| *PRDX5* | *PRF1* | *PRG2* | *PRICKLE1* | *PRKAA2* | *PRKACA* | *PRKACB* | *PRKACG* | *PRKAR1A* | *PRKAR1B* |
| *PRKAR2A* | *PRKAR2B* | *PRKCA* | *PRKCB* | *PRKCD* | *PRKCE* | *PRKCG* | *PRKCQ* | *PRKDC* | *PRKX* |
| *PRL* | *PRLR* | *PRM1* | *PRMT8* | *PROM1* | *PROS1* | *PRPF38A* | *PRR5* | *PRRX1* | *PRSS1* |
| *PRUNE1* | *PSAT1* | *PSEN1* | *PSEN2* | *PSMB10* | *PSMB2* | *PSMB3* | *PSMB5* | *PSMB7* | *PSMB8* |
| *PSMB9* | *PSMC4* | *PSMD7* | *PSPH* | *PTCD2* | *PTCH1* | *PTCH2* | *PTCRA* | *PTEN* | *PTGDR2* |
| *PTGDS* | *PTGER4* | *PTGFRN* | *PTGS2* | *PTK2* | *PTK7* | *PTN* | *PTPN11* | *PTPN5* | *PTPN6* |
| *PTPN7* | *PTPRC* | *PTPRCAP* | *PTPRD* | *PTPRE* | *PTPRN2* | *PTPRR* | *PTPRZ1* | *PTTG1* | *PTTG2* |
| *PUM1* | *PURA* | *PVR* | *PVRIG* | *PWWP2A* | *PYCARD* | *PYCR1* | *PYCR2* | *PYCR3* | *PYGL* |
| *QKI* | *RAB3IL1* | *RAB7A* | *RABGAP1L* | *RAC1* | *RAC2* | *RAC3* | *RAD18* | *RAD21* | *RAD23B* |
| *RAD50* | *RAD51* | *RAD51C* | *RAD52* | *RAD54L* | *RAF1* | *RAG1* | *RALA* | *RALB* | *RALBP1* |
| *RALGDS* | *RANBP2* | *RAP1A* | *RAP1B* | *RAPGEF1* | *RARA* | *RARB* | *RASA4* | *RASAL1* | *RASGEF1B* |
| *RASGRF1* | *RASGRF2* | *RASGRP1* | *RASGRP2* | *RASSF1* | *RASSF5* | *RB1* | *RBL2* | *RBM45* | *RBMS3* |
| *RBP4* | *RBPMS* | *RBX1* | *RCC1* | *REG4* | *REL* | *RELA* | *RELB* | *RELN* | *REN* |
| *REPS1* | *RET* | *REV1* | *REV3L* | *RFC3* | *RFC4* | *RGMB* | *RGS17* | *RHOA* | *RHOB* |
| *RICTOR* | *RIMKLA* | *RIMKLB* | *RIN1* | *RIPK1* | *RIPK2* | *RIPK3* | *RNF130* | *RNF213* | *RNF43* |
| *RNF8* | *RNLS* | *ROBO4* | *ROCK1* | *ROPN1* | *ROR2* | *RORA* | *RORC* | *ROS1* | *RPA3* |
| *RPL23* | *RPL3* | *RPL4* | *RPL7A* | *RPLP0* | *RPS11* | *RPS14* | *RPS27A* | *RPS4Y1* | *RPS6* |
| *RPS6KA5* | *RPS6KA6* | *RPS6KB1* | *RPS6KB2* | *RPS9* | *RPTOR* | *RRAD* | *RRAS2* | *RRM2* | *RRS1* |
| *RSAD2* | *RSPH14* | *RTN4RL1* | *RUNX1* | *RUNX1T1* | *RUNX2* | *RUNX3* | *RXRA* | *RXRB* | *RXRG* |
| *RYBP* | *S100A12* | *S100A2* | *S100A4* | *S100A7* | *S100A8* | *S100A9* | *S100B* | *S100P* | *SAA1* |
| *SAMD9* | *SAMHD1* | *SAMSN1* | *SAP130* | *SARS* | *SBNO2* | *SCGB2A2* | *SCP2* | *SCUBE2* | *SCYL3* |
| *SDC1* | *SDC4* | *SDHA* | *SEC22B* | *SEC31A* | *SEC61G* | *SEL1L3* | *SELE* | *SELENBP1* | *SELENOK* |
| *SELL* | *SELP* | *SELPLG* | *SEMA6A* | *SEMG1* | *SENP1* | *SEPT10* | *SEPT14* | *SEPTIN3* | *SERINC1* |
| *SERINC2* | *SERINC3* | *SERINC5* | *SERPINA1* | *SERPINA3* | *SERPINB2* | *SERPINB3* | *SERPINB5* | *SERPINE1* | *SERPING1* |
| *SERPINH1* | *SETBP1* | *SETD2* | *SF3A1* | *SF3A3* | *SF3B1* | *SFN* | *SFRP1* | *SFRP2* | *SFRP4* |
| *SFTPB* | *SFTPC* | *SFXN1* | *SGK1* | *SGK2* | *SH2B2* | *SH2B3* | *SH2D1A* | *SH2D1B* | *SHC1* |
| *SHC2* | *SHC3* | *SHC4* | *SHH* | *SHROOM3* | *SHTN1* | *SIGIRR* | *SIGLEC1* | *SIGLEC5* | *SIGLEC8* |
| *SIL1* | *SIN3A* | *SIRPA* | *SIRPB2* | *SIRT4* | *SIT1* | *SIX1* | *SKAP2* | *SKP1* | *SKP2* |
| *SLAMF1* | *SLAMF6* | *SLAMF7* | *SLAMF8* | *SLC11A1* | *SLC12A7* | *SLC16A1* | *SLC16A2* | *SLC1A5* | *SLC23A2* |
| *SLC25A1* | *SLC26A4* | *SLC2A1* | *SLC34A2* | *SLC35F2* | *SLC35F3* | *SLC39A6* | *SLC3A1* | *SLC3A2* | *SLC43A1* |
| *SLC43A2* | *SLC45A3* | *SLC4A1AP* | *SLC4A4* | *SLC4A7* | *SLC5A5* | *SLC5A8* | *SLC6A13* | *SLC7A5* | *SLMAP* |
| *SMAD2* | *SMAD3* | *SMAD4* | *SMAD5* | *SMAD9* | *SMAP1* | *SMARCA2* | *SMARCA4* | *SMARCB1* | *SMARCC1* |
| *SMARCC2* | *SMARCD1* | *SMARCD2* | *SMARCD3* | *SMARCE1* | *SMC1A* | *SMC1B* | *SMC3* | *SMO* | *SMPD3* |
| *SNAI1* | *SNAI2* | *SNCA* | *SND1* | *SOCS1* | *SOCS2* | *SOCS3* | *SOD1* | *SOD2* | *SORBS1* |
| *SORBS2* | *SOS1* | *SOS2* | *SOST* | *SOX10* | *SOX11* | *SOX17* | *SOX2* | *SOX4* | *SOX9* |
| *SP1* | *SPA17* | *SPACA3* | *SPAG17* | *SPANXB1* | *SPECC1L* | *SPI1* | *SPIB* | *SPINK1* | *SPINK5* |
| *SPINT1* | *SPN* | *SPO11* | *SPOCK2* | *SPOP* | *SPP1* | *SPRED1* | *SPRED2* | *SPRY1* | *SPRY2* |
| *SPRY4* | *SQSTM1* | *SRC* | *SRD5A2* | *SREBF1* | *SRGN* | *SRP54* | *SRR* | *SRSF2* | *SS18* |
| *SSBP1* | *SSBP2* | *SST* | *SSX1* | *SSX2* | *SSX4* | *ST6GAL1* | *ST7* | *STAG2* | *STARD3* |
| *STAT1* | *STAT2* | *STAT3* | *STAT4* | *STAT5A* | *STAT5B* | *STAT6* | *STC1* | *STING1* | *STK11* |
| *STK11IP* | *STK17B* | *STK26* | *STK4* | *STMN1* | *STMN2* | *STON1-GTF2A1L* | *STRN* | *STRN3* | *SUFU* |
| *SULF1* | *SULT2A1* | *SUMO1* | *SUV39H2* | *SYCP1* | *SYK* | *SYT12* | *SYT17* | *TAB1* | *TACC1* |
| *TACC2* | *TACC3* | *TACSTD2* | *TAF3* | *TAGAP* | *TAL1* | *TANK* | *TAP1* | *TAP2* | *TAPBP* |
| *TAPBPL* | *TARP* | *TATDN1* | *TAX1BP1* | *TBC1D1* | *TBC1D10B* | *TBC1D2* | *TBK1* | *TBL1XR1* | *TBP* |
| *TBX21* | *TBXAS1* | *TCF3* | *TCF7* | *TCF7L1* | *TCF7L2* | *TCIM* | *TCL1A* | *TCL1B* | *TDO2* |
| *TEAD2* | *TECR* | *TERC* | *TERF2* | *TERT* | *TET2* | *TFDP1* | *TFE3* | *TFEB* | *TFG* |
| *TFRC* | *TG* | *TGFA* | *TGFB1* | *TGFB2* | *TGFB3* | *TGFBR1* | *TGFBR2* | *TH* | *THBD* |
| *THBS1* | *THBS4* | *THEM4* | *THRA* | *THRB* | *THY1* | *TIAM1* | *TICAM1* | *TICAM2* | *TIE1* |
| *TIGIT* | *TIRAP* | *TLCD2* | *TLE4* | *TLE5* | *TLK2* | *TLR1* | *TLR10* | *TLR2* | *TLR3* |
| *TLR4* | *TLR5* | *TLR6* | *TLR7* | *TLR8* | *TLR9* | *TLX1* | *TM4SF4* | *TMEFF2* | *TMEM106B* |
| *TMEM140* | *TMEM163* | *TMEM165* | *TMEM43* | *TMEM45B* | *TMPRSS2* | *TMPRSS3* | *TMPRSS4* | *TMUB2* | *TNC* |
| *TNF* | *TNFAIP3* | *TNFAIP6* | *TNFAIP8* | *TNFRSF10A* | *TNFRSF10B* | *TNFRSF10C* | *TNFRSF10D* | *TNFRSF11A* | *TNFRSF11B* |
| *TNFRSF12A* | *TNFRSF13B* | *TNFRSF13C* | *TNFRSF14* | *TNFRSF17* | *TNFRSF18* | *TNFRSF19* | *TNFRSF1A* | *TNFRSF1B* | *TNFRSF25* |
| *TNFRSF4* | *TNFRSF6B* | *TNFRSF8* | *TNFRSF9* | *TNFSF10* | *TNFSF11* | *TNFSF12* | *TNFSF13* | *TNFSF13B* | *TNFSF14* |
| *TNFSF15* | *TNFSF18* | *TNFSF4* | *TNFSF8* | *TNFSF9* | *TNKS* | *TNN* | *TNR* | *TOLLIP* | *TOP2A* |
| *TOX* | *TP53* | *TP63* | *TP73* | *TPD52L1* | *TPI1* | *TPM1* | *TPM2* | *TPM3* | *TPM4* |
| *TPO* | *TPR* | *TPSAB1* | *TPSB2* | *TPTE* | *TPX2* | *TRAF1* | *TRAF2* | *TRAF3* | *TRAF4* |
| *TRAF5* | *TRAF6* | *TRAF7* | *TRAK1* | *TRAT1* | *TREM1* | *TREM2* | *TRIM15* | *TRIM21* | *TRIM24* |
| *TRIM27* | *TRIM29* | *TRIM33* | *TRIM39* | *TRIM63* | *TSC1* | *TSC2* | *TSHR* | *TSLP* | *TSPAN7* |
| *TSPAN8* | *TTC30A* | *TTC31* | *TTK* | *TTPA* | *TTR* | *TUBB* | *TUSC3* | *TWF1* | *TWIST1* |
| *TWIST2* | *TXK* | *TXLNA* | *TXLNGY* | *TXN2* | *TXNIP* | *TXNRD1* | *TXNRD2* | *TXNRD3* | *TYK2* |
| *TYMP* | *TYMS* | *TYROBP* | *TYRP1* | *U2AF1* | *UBA7* | *UBB* | *UBC* | *UBE2C* | *UBE2T* |
| *ULBP2* | *UNC5D* | *UNG* | *UPK1B* | *UPK3A* | *UQCR10* | *UQCR11* | *UQCRQ* | *USP10* | *USP39* |
| *USP8* | *USP9Y* | *UST* | *UTY* | *VCAM1* | *VCAN* | *VCL* | *VEGFA* | *VEGFB* | *VEGFC* |
| *VEGFD* | *VHL* | *VIM* | *VOPP1* | *VPS33B* | *VSIR* | *VSTM2A* | *VTCN1* | *WAC* | *WDCP* |
| *WDR3* | *WDR76* | *WEE1* | *WIF1* | *WIPF1* | *WIPF2* | *WNK2* | *WNT1* | *WNT10A* | *WNT10B* |
| *WNT11* | *WNT16* | *WNT2* | *WNT2B* | *WNT3* | *WNT3A* | *WNT4* | *WNT5A* | *WNT5B* | *WNT6* |
| *WNT7A* | *WNT7B* | *WNT8A* | *WNT8B* | *WNT9A* | *WNT9B* | *WRN* | *WT1* | *WWC1* | *XAGE1B* |
| *XCL1* | *XCL2* | *XCR1* | *XIAP* | *XIST* | *XPA* | *XRCC2* | *XRCC4* | *XRCC5* | *XRCC6* |
| *XXYLT1* | *YRDC* | *YTHDF2* | *YWHAE* | *ZAN* | *ZAP70* | *ZBTB16* | *ZBTB17* | *ZBTB20* | *ZBTB32* |
| *ZBTB46* | *ZC3H12A* | *ZC3H14* | *ZC3HAV1* | *ZCCHC8* | *ZEB1* | *ZEB2* | *ZIC2* | *ZKSCAN5* | *ZMYM2* |
| *ZMYM4* | *ZNF143* | *ZNF205* | *ZNF34* | *ZNF346* | *ZNF365* | *ZNF384* | *ZNF485* | *ZNF703* | *ZSCAN30* |
